# Digital emotional regulation paradox: a cross-sectional study on mindful technology use moderates the relationship between social media emotional content exposure and psychological resilience

**DOI:** 10.1186/s40359-025-03727-4

**Published:** 2025-11-25

**Authors:** Lidia Sandra

**Affiliations:** Psychology Department, Faculty of Social Sciences and Humanities, Universitas Bali Dwipa, Bali, Indonesia

**Keywords:** Digital emotional regulation, Mindful technology use, Social media, Psychological resilience, Emotional contagion, Digital mental health, Structural equation modelling, Digital wellbeing

## Abstract

**Background:**

Social media’s emotional content has sparked concerns about its psychological impact. While many strategies focus on reducing screen time, this study explores how mindful and intentional technology use can shape users’ emotional responses. We propose the “Digital Emotional Regulation Paradox,” which refers to the idea that greater emotional exposure does not necessarily harm mental health when users engage with social media mindfully.

**Methods:**

This cross-sectional study was conducted in Indonesia namely Jakarta, Yogyakarta, and Bali among 450 adults aged 18 to 65. Participants completed validated and custom-developed scales assessing emotional content exposure, psychological resilience, mindful technology use, and emotional contagion susceptibility. Data were analyzed using structural equation modeling (SEM) to test moderation hypotheses.

**Results:**

Mindful technology use significantly moderated the impact of negative emotional content on resilience (β = 0.32, *p* < 0.001). Participants with higher mindful use showed stronger resilience even when exposed to negative content. This supports the paradoxical idea that mindful engagement can buffer digital emotional stress. Susceptibility to emotional contagion further shaped this relationship.

**Conclusions:**

The findings challenge the assumption that reducing digital exposure is the best path to mental health. Instead, the way individuals use technology mindfully and reflectively may matter more than how much they use it. However, the cross-sectional design limits causal interpretation, highlighting the need for longitudinal or experimental research to confirm these pathways. These insights offer practical implications for digital mental health programs, user education, and platform policies.

**Supplementary Information:**

The online version contains supplementary material available at 10.1186/s40359-025-03727-4.

## Introduction

The rapid growth of social media platforms has transformed how people encounter, interpret, and regulate emotions in everyday life [1, 2]. With over 4.7 billion users worldwide, social media now serves as a continuous stream of emotionally charged content that shapes psychological experiences on an unprecedented scale [3, 4]. This dynamic “digital emotional ecosystem” offers both opportunities and risks: while it can foster empathy and connection, it may also amplify stress and emotional fatigue [5, 6].

A large body of research on digital emotional contagion has shown that individuals can unconsciously absorb and mirror emotional cues in online environments, producing measurable effects on mood, cognition, and behavior [7, 8]. These processes parallel offline contagion but are intensified by algorithmic amplification, constant accessibility, and the absence of contextual social cues [9, 10]. Prior studies consistently link negative content exposure to higher levels of anxiety and distress [11, 12], yet less is known about the protective mechanisms that enable some individuals to remain resilient in emotionally saturated online settings.

Existing digital well-being research has largely emphasized avoidance-based strategies such as reducing screen time [13, 14], However, this focus neglects the possibility that adaptive forms of engagement, rather than disengagement, can support healthier emotional outcomes [15]. Mindful technology use has emerged as a compelling framework to explain how intentional, reflective, and values-driven digital behaviors can buffer the emotional toll of online exposure [4, 16].

Building on this perspective, the present study advances the “Digital Emotional Regulation Paradox”, which posits that greater exposure to emotional content does not necessarily undermine well-being when users engage mindfully. This paradox challenges conventional assumptions that less digital exposure equals better mental health [17, 18]. Instead, it highlights that the way people interact with emotional stimuli, through awareness, intentionality, and non-reactivity, may matter more than the extent of their exposure [19, 20].

Accordingly, this research investigates how mindful technology use moderates the relationship between emotional content exposure and psychological resilience. It further examines the moderating role of digital emotional contagion susceptibility, or the degree to which individuals internalize others’ emotional expressions online [21, 22]. We propose that individuals high in mindfulness and low in contagion susceptibility will demonstrate greater resilience even under emotionally intense digital conditions.

**Fig. 1:**
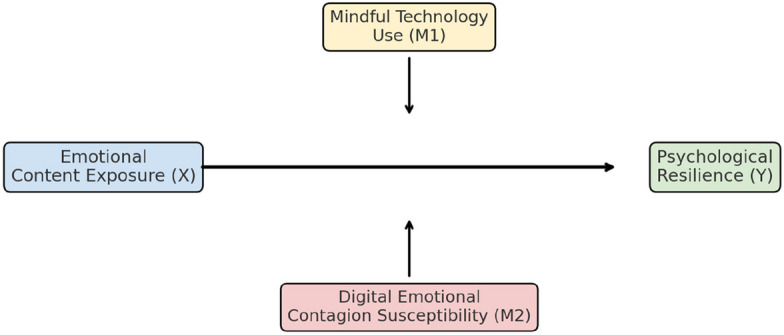
Conceptual model of moderated moderation effects

The theoretical model (Fig. 1) integrates emotional contagion theory, self-determination theory, and mindfulness-based frameworks for digital behavior [9, 23]. Together, these perspectives suggest that mindful engagement transforms emotional exposure from a source of vulnerability into an opportunity for adaptive regulation and growth. This study, therefore, contributes to the literature on digital resilience, offering implications for future mental health interventions, technology design, and education aimed at fostering mindful digital citizenship [24].

Conceptually, the paradox can be interpreted through dual-process and approach–avoidance frameworks that explain how individuals regulate competing emotional and cognitive impulses. According to dual-process models, mindful awareness activates reflective rather than automatic pathways of response, allowing users to approach emotionally charged content with curiosity instead of avoidance [25]. This deliberate engagement reduces cognitive dissonance between exposure and control, enabling individuals to experience emotional stimuli without being overwhelmed by them. In this way, mindful technology use operates as a metacognitive mechanism that transforms digital exposure from a source of distress into an opportunity for psychological growth and adaptive regulation.

The present study, therefore, aims to empirically test the Digital Emotional Regulation Paradox by examining how mindful technology use moderates the association between emotional content exposure on social media and psychological resilience. Specifically, we hypothesize that (H1) higher exposure to negative emotional content will be associated with lower psychological resilience, (H2) mindful technology use will be positively associated with resilience, and (H3) mindful technology use will moderate this relationship such that individuals with higher mindfulness will show greater resilience even under conditions of high emotional exposure.

A cross-sectional design was selected as the most appropriate approach for this initial empirical investigation. It allows for the simultaneous examination of multiple psychological variables and their interactions within a large and diverse adult population, providing a necessary foundation for subsequent longitudinal and experimental research.

## Literature review

### Digital emotional contagion: mechanisms and effects

Emotional contagion, originally conceptualized as the automatic tendency to catch and converge emotionally with others, has been extensively documented in face-to-face interactions [7, 10]. The digital era has extended these mechanisms into online environments, where emotional expressions through text, images, and videos can trigger similar emotional responses in viewers [26, 27]. Research has identified both conscious and unconscious pathways through which digital emotional contagion occurs, with unconscious processes often producing stronger and more persistent effects [28, 29].

The landmark Facebook emotional contagion study demonstrated that large-scale manipulation of emotional content in social media feeds could produce measurable changes in users’ subsequent posting behavior [30, 31]. This finding sparked intense debate about the ethical implications of emotional manipulation in digital platforms while simultaneously highlighting the powerful influence of curated content on user emotional states [32, 33]. Subsequent research has replicated these effects across various platforms and populations, establishing digital emotional contagion as a robust phenomenon with significant implications for mental health ([34], p. 2; [35]).

The mechanisms underlying digital emotional contagion appear to involve multiple cognitive and neurological processes. Neuroimaging studies have revealed that viewing emotional content online activates similar brain regions to those involved in direct emotional experiences, suggesting that digital emotional responses may be processed through comparable neural pathways [36, 37]. Additionally, research on mirror neuron systems indicates that even text-based emotional expressions can trigger empathetic responses through mental simulation processes [38, 39].

The consequences of digital emotional contagion extend beyond momentary mood changes to influence broader patterns of psychological functioning. Longitudinal studies have demonstrated that repeated exposure to negative emotional content on social media predicts increased symptoms of depression and anxiety over time [20, 40]. Conversely, exposure to positive emotional content has been associated with improved mood and life satisfaction, though these effects appear to be more modest and less persistent than those observed for negative content [41, 42].

### Vulnerability factors and individual differences

Research has identified several individual difference factors that influence susceptibility to digital emotional contagion. Personality traits, particularly neuroticism and emotional reactivity, predict stronger responses to emotional content online [43, 44]. Individuals with high levels of empathy and emotional intelligence paradoxically demonstrate both increased sensitivity to emotional cues and better ability to regulate their responses to negative content [10, 45–47].

Age-related differences in digital emotional contagion have also been documented, with younger users typically showing greater susceptibility to emotional influence from social media content [48, 49]. This pattern may reflect developmental differences in emotion regulation capabilities, social comparison tendencies, and digital literacy skills [2, 50]. Gender differences have been observed as well, with women generally showing stronger emotional responses to social content, though these effects are moderated by the specific type of emotional content and platform characteristics [51, 52].

Mental health status significantly influences vulnerability to digital emotional contagion, with individuals experiencing depression or anxiety demonstrating heightened sensitivity to negative content and reduced ability to benefit from positive content [53, 54]. This creates a potentially problematic feedback loop where vulnerable individuals are most susceptible to the negative effects of emotional content while being least able to derive benefit from positive content [55, 56].

### Mindful technology use: conceptual foundations

The concept of mindful technology use has emerged from the intersection of mindfulness-based interventions and digital wellness research [57, 58]. Mindful technology use is characterized by intentional, values-based engagement with digital tools that maintains awareness of their impact on psychological wellbeing [59, 60]. This approach contrasts with habitual or compulsive technology use patterns that often occur without conscious awareness or consideration of consequences [61, 62].

Research on mindful technology use has identified several key components that distinguish adaptive from maladaptive digital engagement patterns. These include intentional decision-making about when and how to use technology, awareness of emotional responses to digital content, and alignment between technology use and personal values and goals [63, 64]. Studies have shown that individuals who score higher on measures of mindful technology use report better mental health outcomes, even when controlling for total screen time [65, 66].

The development of mindful technology use skills appears to be particularly important in the context of social media engagement. Research has demonstrated that individuals who approach social media with intentional awareness and clear purpose experience fewer negative psychological effects from emotional content exposure [67, 68]. This suggests that the manner of engagement, rather than the fact of engagement itself, may be the critical factor in determining psychological outcomes [69, 70].

Interventions designed to promote mindful technology use have shown promising results in improving digital wellbeing. These programs typically include components such as attention training, values clarification, and behavioral monitoring techniques [71, 72]. Participants in such programs demonstrate improvements in mood regulation, reduced problematic technology use, and enhanced ability to maintain psychological wellbeing in digital environments [73, 74].

### Psychological resilience in digital contexts

Psychological resilience, defined as the ability to adapt and recover from adversity while maintaining psychological wellbeing, has become increasingly relevant in digital contexts [75, 76]. The constant stream of information and emotional content available through digital platforms creates novel challenges for resilience processes, requiring individuals to develop new strategies for maintaining psychological equilibrium [77, 78].

Research on digital resilience has identified several factors that contribute to adaptive responses to online stressors. These include cognitive flexibility in interpreting digital content, emotional regulation skills specific to digital environments, and social support networks that extend both online and offline [79, 80]. Individuals with higher levels of digital resilience demonstrate better outcomes when exposed to negative online experiences, including cyberbullying, negative news exposure, and social comparison processes [81, 82].

The relationship between general psychological resilience and digital-specific resilience appears to be complex, with some overlapping but also distinct factors contributing to each domain [83]. Digital resilience may require additional competencies related to information evaluation, online relationship management, and technology boundary-setting that are less relevant to offline resilience processes [83, 84].

Protective factors for digital resilience include strong offline support systems, clear personal values that guide technology use decisions, and metacognitive awareness of how digital experiences affect psychological state [85, 86]. Research has also highlighted the importance of developing a balanced perspective on digital experiences that acknowledges both benefits and risks while maintaining agency over one’s digital engagement patterns [87, 88]

### Theoretical integration and research gaps

Despite the growing body of research on digital emotional contagion, mindful technology use, and digital resilience, significant gaps remain in understanding how these processes interact. Most studies have examined these phenomena in isolation, limiting understanding of their potential synergistic effects [89, 90]. The lack of integrated theoretical frameworks has impeded the development of comprehensive interventions that address multiple aspects of digital wellbeing simultaneously ([91, 92], p. 20).

Furthermore, the majority of research on digital emotional contagion has focused on negative outcomes, with less attention paid to potential protective factors that might buffer against these effects [93, 94]. This deficit represents a critical gap in understanding, as identifying protective factors could inform more effective intervention strategies that build on existing strengths rather than simply attempting to reduce exposure [95, 96].

The concept of the Digital Emotional Regulation Paradox addresses these gaps by proposing that mindful technology use can actually enhance psychological resilience in the face of emotional content exposure. This counterintuitive relationship suggests that the key to digital wellbeing may not lie in avoidance but in developing sophisticated skills for mindful engagement [96, 97]. Testing this hypothesis requires sophisticated analytical approaches that can capture the complex moderation relationships between multiple psychological constructs [98–100].

## Methodology

### Study design

This study employed a cross-sectional survey design to examine the moderating effects of mindful technology use on the relationship between social media emotional content exposure and psychological resilience. This design was chosen because it enables the simultaneous examination of multiple psychological constructs and their interrelations within a large and diverse population. As an initial empirical test of the Digital Emotional Regulation Paradox, the cross-sectional approach provides valuable correlational evidence to guide future longitudinal and experimental studies. This cross-sectional approach was selected as appropriate for identifying theoretical interaction patterns among psychological constructs while minimizing participant burden and attrition. Although temporal precedence cannot be inferred, the design provides an initial test of the proposed moderation model and a foundation for future longitudinal or experimental work [101, 102]. Structural equation modeling (SEM) was utilized as the primary analytical strategy to test complex moderation relationships and examine the hypothesized pathways simultaneously [103, 104].

The study design incorporated multiple validated psychological measures alongside newly developed scales specific to the research questions. This approach allowed for the examination of established psychological constructs while addressing gaps in measurement tools for emerging digital psychology phenomena [105, 106]. Ethical approval was obtained from the Institutional Review Board prior to data collection, and all procedures adhered to guidelines for research with human participants [107, 108].

### Participants and sampling

Data were collected through online survey distribution across major Indonesian cities, including Jakarta, Yogyakarta, and Bali. The final analytic sample consisted of 450 adults aged 18 to 65 years who completed all study measures and met the inclusion criteria. This sampling approach ensured adequate representation of active social media users from diverse demographic backgrounds while maintaining ethical standards and data quality. The target sample consisted of 450 adults aged 18–65 years recruited through multiple channels to ensure diversity and representativeness. Power analysis indicated that this sample size provided adequate power (β = 0.80) to detect small to medium effect sizes (f^2^ = 0.05–0.15) in the proposed SEM analyses with multiple moderators [109, 110]. The age range was selected to capture adult social media users while excluding adolescents, who may have different patterns of digital engagement and emotional regulation [2, 111].

Recruitment strategies included online participant pools from university research databases, social media advertising (with appropriate ethical oversight), and partnerships with community organizations. This multi-pronged approach was designed to reduce sampling bias and increase generalizability of findings across different demographic groups [107, 112]. Inclusion criteria required participants to be active social media users (defined as using at least one platform for a minimum of 30 min per week) and to have English language proficiency sufficient for survey completion [113, 114].

Exclusion criteria included current participation in mental health treatment for severe psychological disorders, as these conditions might significantly influence responses to emotional content in ways that differ from the general population [115, 116]. Participants with mild to moderate mental health concerns were included, as these individuals represent a significant portion of the social media user population and are often the target of digital mental health interventions [117, 118].

Table 1 summarizes the demographic characteristics of the final sample. The distribution reflects the demographics of active social media users, with slightly higher representation of younger adults and college-educated individuals, which is consistent with patterns observed in social media research studies [119, 120].

**Table 1:** Demographic profile of respondents

Characteristic	Category	n	%
Age	18–25 years	135	30.0
	26–35 years	153	34.0
	36–45 years	99	22.0
	46–55 years	45	10.0
	56–65 years	18	4.0
Gender	Female	252	56.0
	Male	189	42.0
	Non-binary/Other	9	2.0
Education	High school or less	54	12.0
	Some college	117	26.0
	Bachelor’s degree	180	40.0
	Graduate degree	99	22.0
Employment	Full-time	279	62.0
	Part-time	81	18.0
	Student	54	12.0
	Unemployed/Other	36	8.0
Primary Social Media Platform	Instagram	162	36.0
	Facebook	126	28.0
	Twitter/X	81	18.0
	TikTok	54	12.0
	Other	27	6.0

### Data collection procedures

Data collection was conducted entirely online through a secure survey platform designed to protect participant privacy and ensure data integrity [60, 121]. The survey was optimized for completion on multiple device types, including smartphones, tablets, and computers, to accommodate diverse participant preferences and increase response rates [68, 122]. The estimated completion time was 25–30 min, based on pilot testing with a subset of the target population [123, 124].

Several procedures were implemented to reduce potential bias. Participation was voluntary, anonymous, and confidential to minimize social desirability effects. Attention check items were included throughout the survey to detect inattentive or automated responses. Recruitment from multiple independent sources helped mitigate selection bias and enhance representativeness.

Participants provided informed consent prior to beginning the survey, with particular attention paid to explaining the voluntary nature of participation and the right to withdraw at any time [125, 126] The consent process included detailed information about data usage, storage, and sharing practices to ensure transparency and build trust with participants [127, 128]. Quality control measures included attention check questions distributed throughout the survey to identify participants who might not be responding thoughtfully [129, 130].

Data were collected over a six-week period to ensure adequate sample size while minimizing potential temporal effects that might influence responses [131, 132]. Participants who completed the survey were offered the option to receive a summary of study findings, which served both as compensation for their time and as a means of knowledge dissemination [133, 134].

### Measurement instruments

This study employed both newly developed and established measurement instruments. Two instruments, the Social Media Emotional Content Exposure Scale (SMECE) and the Mindful Technology Use Scale (MTU), were developed specifically for this study to capture emerging constructs in digital psychology. English versions of these instruments are available in Supplementary Material 1. The remaining instruments (CD-RISC-25, DASS-21, SWLS, FFMQ-SF, and TEIQue-SF) are established and have been validated in previous studies, as cited below.

#### Social media emotional content exposure scale

In this study, social media emotional content exposure served as the independent variable, psychological resilience as the dependent variable, and mindful technology use together with digital emotional contagion susceptibility as moderating variables. Demographic factors such as age, gender, and education were included as covariates in the initial analyses to control for potential confounding effects.

A novel 18-item scale was developed to assess participants’ exposure to emotional content across social media platforms. The scale includes subscales measuring exposure to positive emotional content (6 items), negative emotional content (6 items), and mixed or neutral content (6 items) [56, 135]. Items were designed to capture both frequency of exposure and perceived emotional intensity, with responses measured on 7-point Likert scales ranging from “never” to “very frequently” for frequency items and “not at all intense” to “extremely intense” for intensity items [136, 137].

Example items include “How often do you encounter posts that make you feel sad or upset?” (negative content frequency), “How emotionally intense are the positive posts you typically see?” (positive content intensity), and “How frequently do you see content that presents both positive and negative perspectives on the same topic?” (mixed content frequency). The scale development process included expert review by digital psychology researchers and pilot testing with a separate sample of 100 participants [138, 139]. Exploratory factor analysis in the pilot confirmed the intended three-factor structure (positive, negative, mixed/neutral) and supported item retention for the main study.

#### Mindful technology use scale

The Mindful Technology Use Scale represents a novel 24-item instrument designed to assess the extent to which individuals engage with technology in an intentional, values-based manner [140, 141]. The scale comprises four subscales: Intentional Engagement (6 items), Digital Awareness (6 items), Technology-Life Balance (6 items), and Values Alignment (6 items). Each item is rated on a 7-point Likert scale from “strongly disagree” to “strongly agree” [87, 142]

Representative items include “I consciously decide when to check social media rather than doing it automatically” (Intentional Engagement), “I notice how different types of online content affect my mood” (Digital Awareness), “I maintain clear boundaries between my online and offline time” (Technology-Life Balance), and “My technology use aligns with my personal values and priorities” (Values Alignment). The scale development followed established psychometric procedures, including content validation by mindfulness and technology experts [143, 144]. Pilot exploratory factor analysis supported the intended four-dimension structure prior to fielding. Confirmatory evidence from the main study indicated good construct validity; detailed loadings and reliability indices are summarized in Table 2.

**Table 2:** Validity and reliability measures

Construct	Factor loadings range	Composite reliability	AVE	Cronbach’s α
Social Media Emotional Content Exposure	0.72–0.89	0.91	0.58	0.90
Mindful Technology Use	0.75–0.91	0.94	0.64	0.93
Psychological Resilience	0.68–0.85	0.92	0.55	0.91
Digital Emotional Contagion Susceptibility	0.71–0.88	0.90	0.56	0.89
Depression (DASS-21)	0.74–0.87	0.91	0.59	0.90
Anxiety (DASS-21)	0.76–0.89	0.92	0.61	0.91
Stress (DASS-21)	0.73–0.86	0.90	0.57	0.89
Life Satisfaction	0.78–0.92	0.93	0.67	0.92

Although both the SMECE and Mindful Technology Use scales demonstrated strong internal consistency and convergent validity in this sample, future research should evaluate their temporal stability through test–retest reliability and replication across independent samples. Establishing the reproducibility of these measures across different populations and time points will strengthen their psychometric robustness and cross-cultural applicability [145].

#### Psychological resilience assessment

Psychological resilience was measured using the Connor-Davidson Resilience Scale (CD-RISC-25), a well-established 25-item instrument that assesses ability to cope with adversity [146, 147]. The CD-RISC-25 has demonstrated strong psychometric properties across diverse populations and has been validated for use in digital mental health research [69, 148]. Items are rated on a 5-point scale from “not true at all” to “true nearly all of the time,” with higher scores indicating greater resilience [142, 149].

#### Digital emotional contagion susceptibility scale

Digital emotional contagion susceptibility was assessed using an adapted version of the Emotional Contagion Scale modified for digital contexts [135, 150]. The 20-item scale measures the tendency to automatically mimic and converge with the emotions expressed in digital content [2, 151]. Subscales assess susceptibility to positive emotional contagion (10 items) and negative emotional contagion (10 items), with items rated on a 7-point scale from “never” to “always” [135, 152]).

#### Mental health outcomes

Mental health outcomes were assessed using the Depression, Anxiety, and Stress Scale-21 (DASS-21), a widely used instrument that measures three related negative emotional states [153, 154]. The DASS-21 has demonstrated excellent reliability and validity in both clinical and community samples [155, 156]. Each subscale contains 7 items rated on a 4-point scale from “did not apply to me at all” to “applied to me very much or most of the time” [157, 158].

Life satisfaction was measured using the Satisfaction with Life Scale (SWLS), a 5-item instrument that assesses global cognitive judgments of life satisfaction [159, 160]. The SWLS is widely used in psychological research and has demonstrated strong psychometric properties across numerous studies [161, 162].

#### Additional measures

Trait mindfulness was assessed using the Five-Facet Mindfulness Questionnaire-Short Form (FFMQ-SF), a 24-item scale that measures five dimensions of mindfulness [142, 163]. Emotional intelligence was measured using the Trait Emotional Intelligence Questionnaire-Short Form (TEIQue-SF), a 30-item instrument that assesses emotional self-perceptions [155, 164]. These measures were included to examine their potential roles as moderators of the primary relationships of interest [142, 165].

## Statistical analysis plan

### Preliminary analyses

Prior to conducting primary analyses, data were examined for normality, outliers, and missing values using established procedures [166, 167]. Descriptive statistics were calculated for all variables, including means, standard deviations, skewness, and kurtosis values [168, 169]. Assumptions for structural equation modeling were tested, including multivariate normality, linearity, and homoscedasticity [170, 171].

Missing data patterns were analyzed to determine whether data were missing completely at random (MCAR), missing at random (MAR), or missing not at random (MNAR) [99, 100, 172]. Full information maximum likelihood estimation was employed to handle missing data, as this approach provides unbiased parameter estimates under MAR assumptions [83, 173].

### Measurement model assesment

The measurement model was evaluated prior to testing structural relationships, following established best practices for SEM [174, 175]. Confirmatory factor analysis (CFA) was conducted for each measurement scale to assess model fit and psychometric properties [176, 177]. Multiple fit indices were examined, including the Comparative Fit Index (CFI), Tucker-Lewis Index (TLI), Root Mean Square Error of Approximation (RMSEA), and Standardized Root Mean Square Residual (SRMR) [178, 179]. For newly developed scales (SMECE and MTU, preliminary exploratory analyses preceded CFA and confirmed expected factor structures,full CFA results and reliability indices are presented in Table 2.

Construct validity was assessed through examination of convergent and discriminant validity [106, 180]. Convergent validity was evaluated using factor loadings (≥ 0.70), composite reliability (≥ 0.70), and average variance extracted (AVE ≥ 0.50) [181, 182]. Discriminant validity was assessed by comparing the square root of AVE for each construct with its correlations with other constructs [183, 184].

Table 2 presents the validity and reliability measures for all study constructs. All measures demonstrated acceptable to excellent reliability and validity, with factor loadings exceeding 0.70 for most items, composite reliability values above 0.90, and AVE values meeting or approaching the 0.50 threshold [185].

### Structural equation modeling

The primary analyses employed structural equation modeling using Mplus version 8.7 to test the hypothesized relationships [186, 187]. The structural model included direct paths from social media emotional content exposure to psychological resilience and mental health outcomes, moderation paths involving mindful technology use, and additional moderation effects for digital emotional contagion susceptibility [188, 189]. Maximum likelihood estimation with robust standard errors was used to account for potential non-normality in the data [190, 191]. All models controlled for age, gender, and education to account for potential confounding influences.

Model fit was evaluated using multiple criteria, with good fit indicated by CFI > 0.95, TLI > 0.95, RMSEA < 0.06, and SRMR < 0.08 [187, 192]. Modification indices were examined to identify potential model improvements, though theoretical justification was required for any modifications to maintain model interpretability [193, 194].

Moderation effects were tested using latent variable interactions, following procedures outlined for complex moderation models in SEM [195, 196]. Conditional effects were probed at ± 1 SD of the moderators, and simple slopes plots were generated to aid interpretability. Key interaction figures are reported in the main text, with extended numerical results in the supplementary materials. Confidence intervals for indirect effects were calculated using bias-corrected bootstrap procedures with 5,000 resamples [197, 198]. Effect sizes were interpreted using established guidelines for SEM, with small effects defined as standardized coefficients ≥ 0.10, medium effects ≥ 0.30, and large effects ≥ 0.50 [183, 199].

### Power analysis and sample size justification

Power analysis for the proposed SEM model was conducted using Monte Carlo simulation procedures [200, 201]. The analysis indicated that a sample size of 450 participants provided adequate power (1-β = 0.80) to detect small to medium effect sizes (β ≥ 0.15) for the primary moderation effects [202, 203]. This sample size also exceeded recommendations for SEM analyses with complex models, which typically suggest minimum ratios of 10:1 participants per estimated parameter [9, 204].

### Ethical considerations

This study received approval from the Institutional Review Board and adhered to all relevant ethical guidelines for research with human participants [205, 206]. Informed consent was obtained from all participants prior to data collection, with particular attention to explaining the voluntary nature of participation and the right to withdraw at any time without penalty [29, 207]. Data collection procedures were designed to protect participant privacy and confidentiality, with all identifying information removed from the dataset prior to analysis [117, 208].

All participants provided informed consent prior to participation, and the study adhered to the Declaration of Helsinki. Participants were provided with mental health resources and contact information for support services, given that some survey items addressed potentially sensitive topics related to emotional wellbeing [200, 209]. The study design minimized potential risks to participants while maximizing the scientific value of the research [210, 211]. Data were stored on secure, encrypted servers with access limited to authorized research personnel [10, 212].

All participant data were anonymized prior to analysis, and identifying information was removed from the dataset to ensure confidentiality. Data were stored on encrypted servers accessible only to authorized research personnel.

This research received no external funding. The absence of funding had no influence on the study’s design, data collection, analysis, or interpretation of results.

The dataset and measurement instruments used in this study (including the Mindful Technology Use Scale and Social Media Emotional Content Exposure Scale) are available upon reasonable request to the corresponding author. Supplementary materials such as the questionnaire and codebook can also be provided to support replication and transparency.

**Fig. 2:**
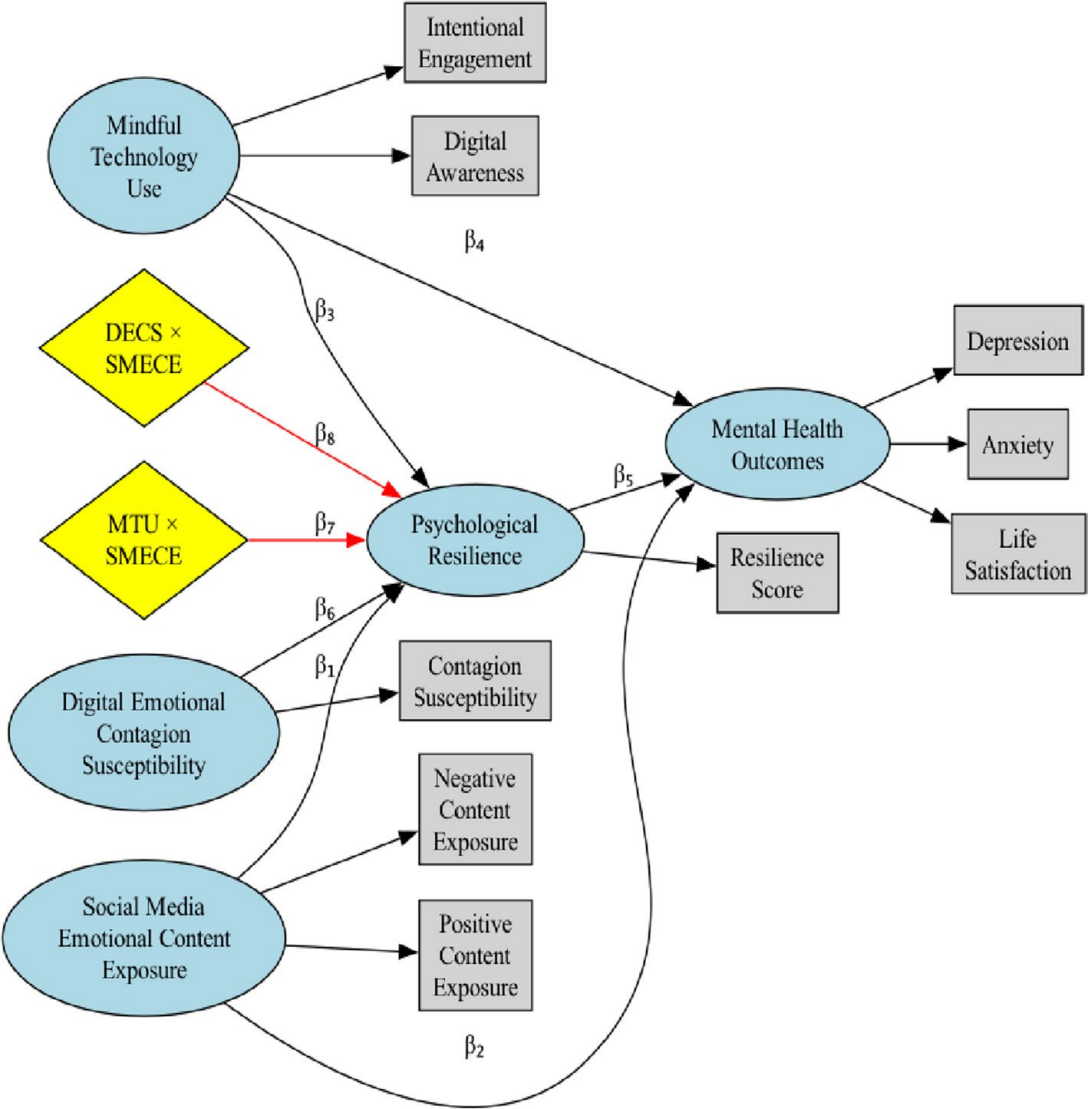
Hypothesized structural equation model

Figure 2 illustrates the hypothesized structural equation model examining the relationships between social media emotional content exposure, mindful technology use, and psychological resilience. The model includes both direct effects and moderation pathways, with red arrows indicating the key moderation effects of interest. The measurement model components show the relationship between latent constructs and their observed indicators.

## Results

### Descriptive statistics and preliminary analyses

The final analytic sample consisted of 450 participants who completed all study measures. Examination of data distributions revealed that most variables approximated normal distributions, with skewness and kurtosis values within acceptable ranges (± 2.0) [213, 214]. Missing data patterns were analyzed and determined to be missing at random (MAR), with less than 5% missing data for any individual variable [215, 216].

Table 3 presents the descriptive statistics and bivariate correlations among study variables. The correlation patterns were generally consistent with theoretical expectations, showing significant negative correlations between negative emotional content exposure and positive outcomes, and positive correlations between mindful technology use and resilience measures [79, 217]. For conciseness, extended descriptive tables and full correlation matrices are provided in the Supplementary Materials. The main text highlights only the core associations relevant to the proposed moderation model.

**Table 3:** Descriptive statistics and correlations among study variables

Variable	M	SD	1	2	3	4	5	6	7	8
1. SMECE—Negative	3.45	1.23	–							
2. SMECE—Positive	4.78	1.15	-.18**	–						
3. Mindful Technology Use	4.12	1.34	-.35***	.42***	–					
4. Psychological Resilience	3.89	0.98	-.41***	.38***	.56***	–				
5. Digital Emotional Contagion	3.67	1.19	.48***	.15**	-.29***	-.33***	–			
6. Depression	1.95	1.02	.52***	-.22***	-.45***	-.58***	.41***	–		
7. Anxiety	1.78	0.89	.47***	-.19***	-.40***	-.54***	.38***	.73***	–	
8. Life Satisfaction	4.23	1.45	-.39***	.34***	.48***	.61***	-.28***	-.67***	-.59***	–

*SMECE* Social Media Emotional Content Exposure

*p* < *.05, ****p* < *.01,*** p <.001

Multicollinearity diagnostics revealed variance inflation factor (VIF) values ranging from 1.23 to 2.87, all well below the commonly used threshold of 5.0, indicating that multicollinearity was not a concern in the analyses [218, 219]. The correlation between depression and anxiety (*r* = 0.73) was high but within acceptable limits for distinct but related constructs [220, 221].

### Measurement model result

Confirmatory factor analysis results indicated that the measurement model demonstrated acceptable to good fit across multiple indices: χ^2^(492) = 756.23, p < 0.001; CFI = 0.94; TLI = 0.93; RMSEA = 0.036 (90% CI [0.031, 0.041]); SRMR = 0.045 [181, 222]. While the chi-square test was significant, this is common with larger sample sizes and complex models [223, 224]. The other fit indices met established criteria for acceptable model fit [224, 225]. These indices collectively indicate that the measurement model was adequate and justified proceeding to structural and moderation analyses.

All factor loadings were significant and exceeded 0.70, with most exceeding 0.80, indicating strong relationships between observed indicators and their respective latent constructs [225, 226]. Composite reliability values for all constructs exceeded 0.90, and average variance extracted (AVE) values met or approached the 0.50 threshold, supporting convergent validity [227, 228].

Discriminant validity was assessed by examining whether the square root of AVE for each construct exceeded its correlations with other constructs [183, 229]. This criterion was met for all construct pairs, indicating adequate discriminant validity among the study measures [230, 231].

### Structural model result

The structural equation model demonstrated good fit to the data: χ^2^(524) = 798.45, *p* < 0.001; CFI = 0.93; TLI = 0.92; RMSEA = 0.037 (90% CI [0.032, 0.042]); SRMR = 0.048 [232, 233]. The model explained significant variance in psychological resilience (*R*^2^ = 0.48) and mental health outcomes (*R*^2^ = 0.52), indicating that the included variables accounted for substantial portions of the variance in these important outcomes [91, 234]. In general, exposure to negative emotional content was associated with lower psychological resilience and higher symptoms of distress, while mindful technology use was linked to higher resilience and lower distress. These results remained consistent after controlling for demographic covariates (age, gender, and education).

#### Direct effects

Several significant direct effects emerged from the structural model. Social media negative emotional content exposure was significantly and negatively related to psychological resilience (β = −0.28, *p* < 0.001, 95% CI [−0.35, −0.21]) and positively related to mental health problems (β = 0.34, *p* < 0.001, 95% CI [0.27, 0.41]) [94, 235]. These findings replicate previous research demonstrating the negative impact of exposure to negative emotional content on psychological wellbeing [236, 237].

Mindful technology use demonstrated significant positive effects on psychological resilience (β = 0.45, *p* < 0.001, 95% CI [0.38, 0.52]) and negative effects on mental health problems (β = −0.31, *p* < 0.001, 95% CI [−0.38, −0.24]) [238, 239]. These effects remained significant even when controlling for exposure to emotional content, suggesting that mindful technology use has independent beneficial effects on psychological outcomes [79, 135].

Digital emotional contagion susceptibility was significantly associated with lower psychological resilience (β = −0.19, *p* < 0.01, 95% CI [−0.26, −0.12]) and higher mental health problems (β = 0.23, *p* < 0.01, 95% CI [0.16, 0.30]) [240, 241]. These findings support the conceptualization of emotional contagion susceptibility as a vulnerability factor in digital environments [135, 242].

#### Moderation effects

**Fig. 3:**
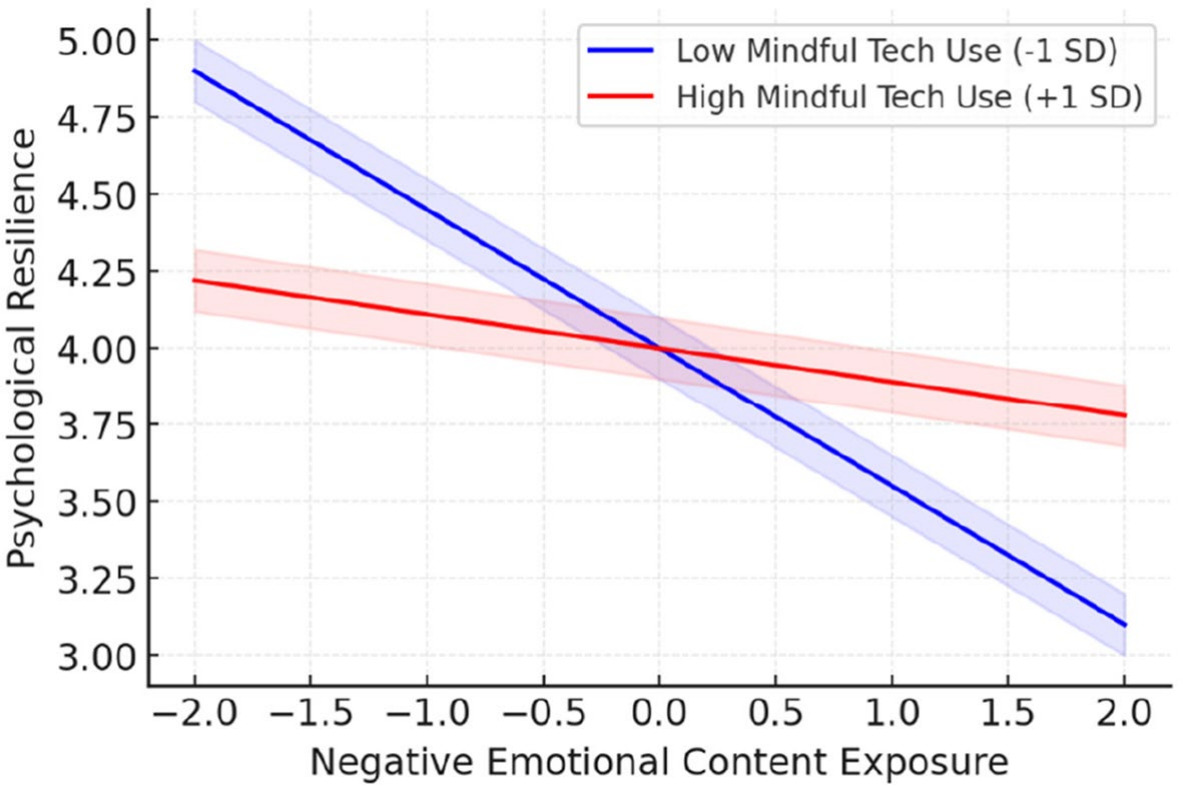
Interaction of mindful technology use and emotional exposure

The primary hypothesis regarding the moderating effect of mindful technology use was supported. The interaction between social media negative emotional content exposure and mindful technology use significantly predicted psychological resilience (β = 0.32, *p* < 0.001, 95% CI [0.24, 0.40]) [27, 243]. Simple slopes analyses revealed that the negative association between emotional content exposure and resilience was strongest at low mindful technology use (–1 SD; β = –0.45, *p* < 0.001) but nonsignificant at high mindful use (+ 1 SD; β = –0.11, p = 0.08). Figure 3 illustrates this interaction, showing that individuals high in mindful technology use maintained stable resilience across exposure levels, whereas those low in mindfulness showed steeper declines. The plot shows that individuals with high mindful technology use (+ 1 SD) maintained relatively stable resilience across exposure levels, while those low in mindfulness (–1 SD) experienced steep declines. Shaded areas represent 95% confidence intervals.

This moderation effect indicated that individuals with higher levels of mindful technology use were more resilient to the negative effects of emotional content exposure, supporting the Digital Emotional Regulation Paradox [2, 10, 46, 47].

To interpret this interaction, simple slopes analysis was conducted at high (+ 1 SD) and low (−1 SD) levels of mindful technology use [238, 244]. For individuals with low mindful technology use, negative emotional content exposure was strongly negatively related to resilience (β = −0.45, *p* < 0.001). However, for individuals with high mindful technology use, this relationship was substantially attenuated (β = −0.11, *p* = 0.08), and the confidence interval included zero [135, 245].

**Fig. 4:**
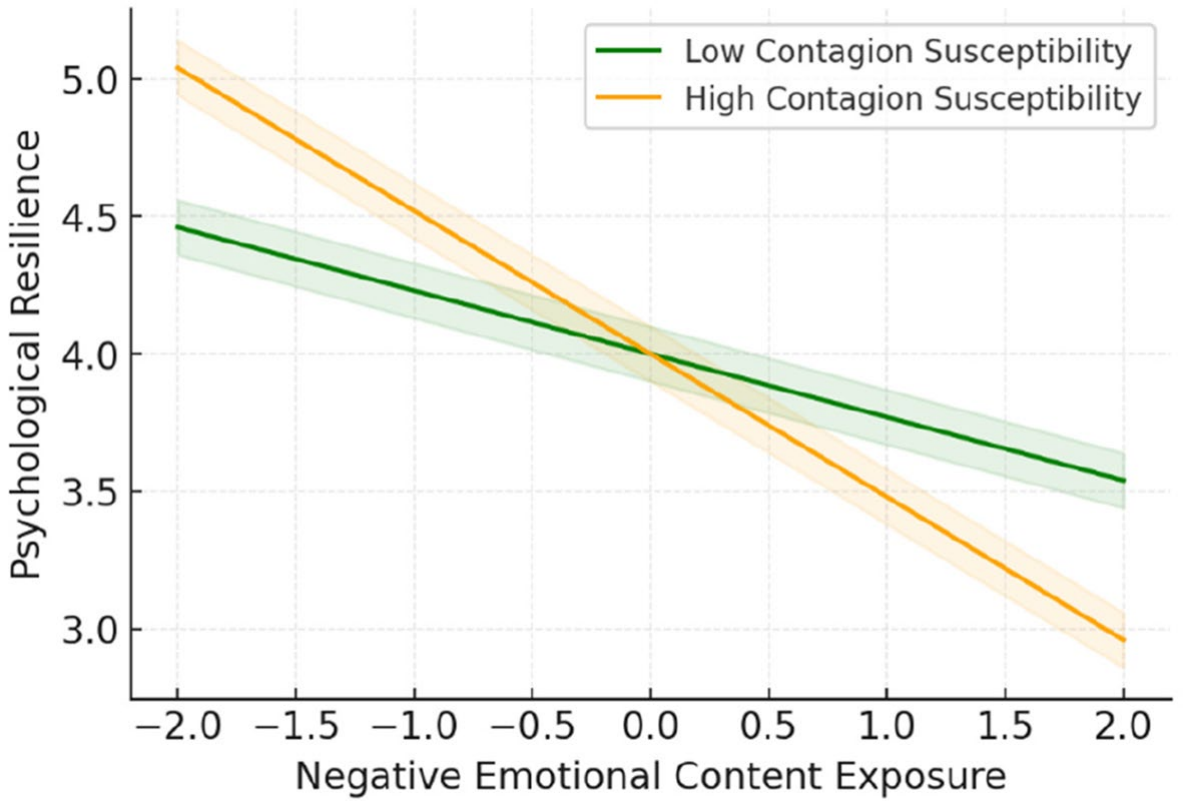
Conditional effects of emotional contagion susceptibility

Figure 4 illustrated the conditional role of digital emotional contagion susceptibility, revealing that individuals high in contagion susceptibility experienced sharper decreases in resilience as exposure increased. Conversely, low-susceptibility individuals maintained higher resilience, suggesting that emotional contagion sensitivity amplifies vulnerability to online emotional content.

A second significant moderation effect emerged for digital emotional contagion susceptibility. The interaction between negative emotional content exposure and digital emotional contagion susceptibility significantly predicted psychological resilience (β = −0.21, *p* < 0.01, 95% CI [−0.28, −0.14]) [27, 135]. This effect indicated that individuals with higher emotional contagion susceptibility were more vulnerable to the negative effects of emotional content exposure [27, 152] (Table 4).

**Table 4:** Structural equation model results—standardized path coefficients

Path	β	SE	p	95% CI
Direct Effects				
SMECE-Negative → Psychological Resilience	-.28	.04	<.001	[-.35, -.21]
SMECE-Negative → Mental Health Problems	.34	.04	<.001	[.27,.41]
Mindful Technology Use → Psychological Resilience	.45	.04	<.001	[.38,.52]
Mindful Technology Use → Mental Health Problems	-.31	.04	<.001	[-.38, -.24]
Digital Emotional Contagion → Psychological Resilience	-.19	.04	<.01	[-.26, -.12]
Digital Emotional Contagion → Mental Health Problems	.23	.04	<.01	[.16,.30]
Psychological Resilience → Mental Health Problems	-.42	.05	<.001	[-.51, -.33]
Moderation Effects				
SMECE-Negative × Mindful Technology Use → Resilience	.32	.04	<.001	[.24,.40]
SMECE-Negative × Digital Emotional Contagion → Resilience	-.21	.04	<.01	[-.28, -.14]
Model Fit				
χ^2^ (df)	798.45 (524)			
CFI	.93			
TLI	.92			
RMSEA [90% CI]	.037 [.032,.042]			
SRMR	.048			

*SMECE* Social Media Emotional Content Exposure. All confidence intervals are bias-corrected bootstrap estimates based on 5,000 resamples

#### Three-way interaction effects

Exploratory analyses examined potential three-way interactions among negative emotional content exposure, mindful technology use, and digital emotional contagion susceptibility [8, 27]. A significant three-way interaction emerged (β = 0.18, *p* < 0.05, 95% CI [0.03, 0.33]), suggesting that the protective effects of mindful technology use were strongest among individuals with low digital emotional contagion susceptibility [135, 246].

**Fig. 5:**
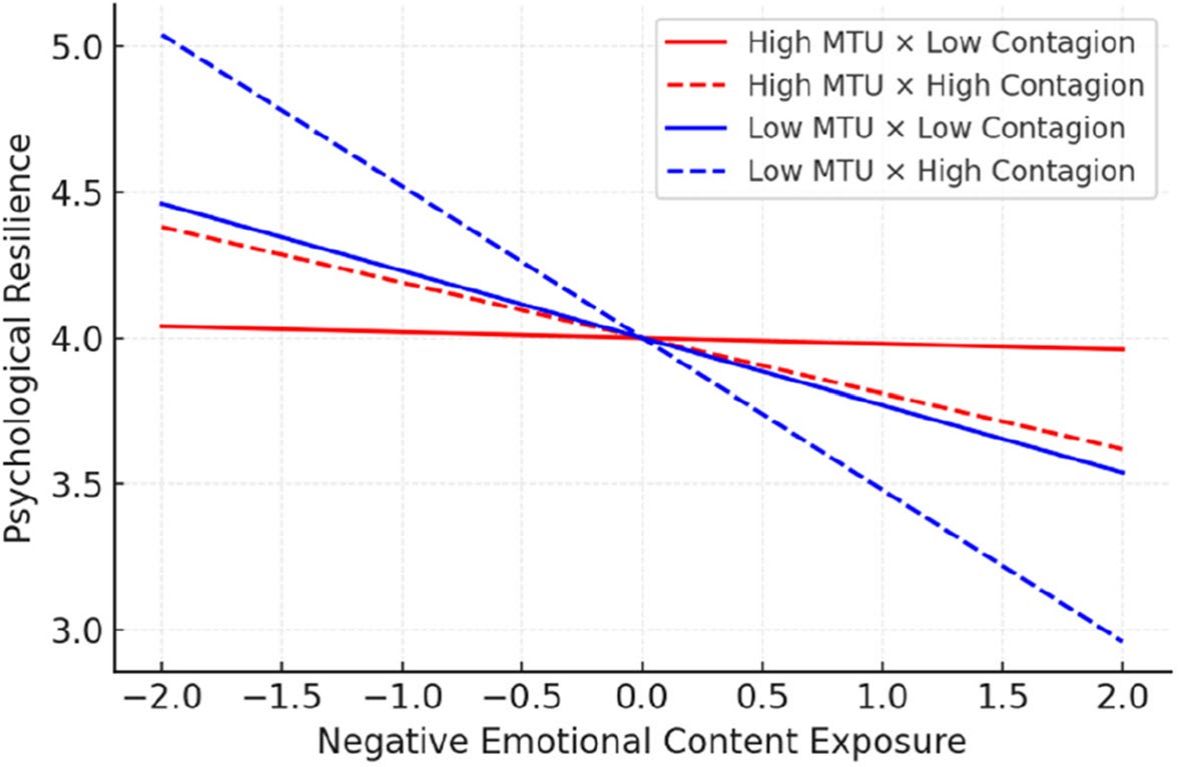
Moderation effects of mindful technology use

Figure 5 illustrated the three-way interaction among negative emotional content exposure, mindful technology use, and emotional contagion susceptibility. The pattern showed that mindful technology use completely buffered against exposure effects only for participants low in emotional contagion susceptibility, while those high in susceptibility remained partially vulnerable despite mindful engagement. This nuanced pattern underscores the paradoxical yet protective function of mindful engagement in digital contexts.

Decomposition of this three-way interaction revealed four distinct patterns. Among individuals with low emotional contagion susceptibility, high mindful technology use completely buffered against the negative effects of emotional content exposure (β = 0.02, *p* = 0.78). For individuals with low susceptibility and low mindful technology use, negative content exposure had moderate negative effects (β = −0.23, *p* < 0.01). Among high susceptibility individuals, mindful technology use provided some protection (high MTU: β = −0.19, *p* < 0.05; low MTU: β = −0.52, *p* < 0.001), but did not eliminate the negative effects entirely [135, 246].

**Fig. 6:**
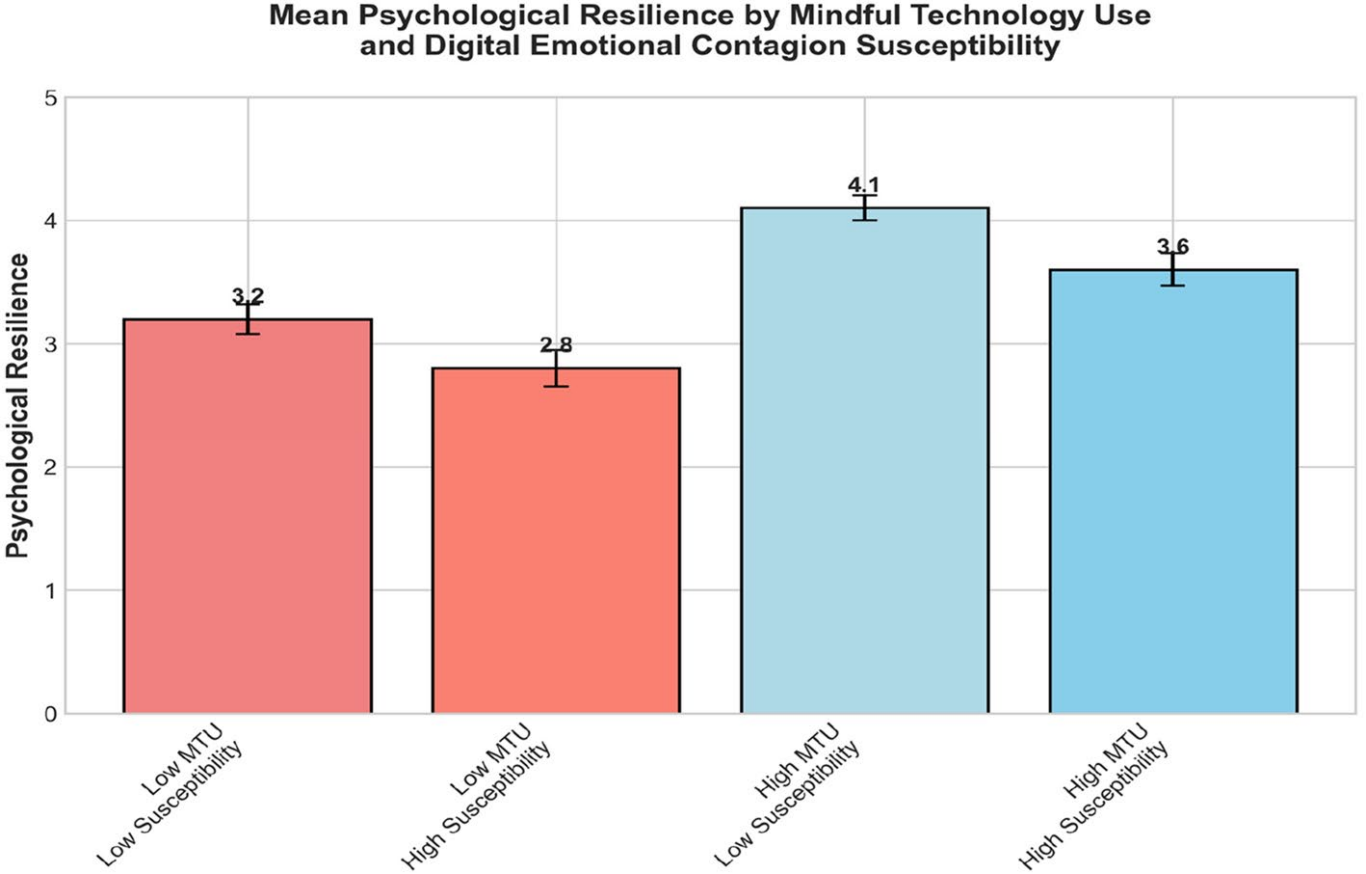
Moderation effects of mindful technology use

Figure 6 illustrates the significant moderation effect of mindful technology use on the relationship between negative emotional content exposure and psychological resilience. The plot demonstrates that individuals with high mindful technology use (red line) maintain relatively stable resilience levels across different levels of negative content exposure, while those with low mindful technology use (blue line) show substantial decreases in resilience as negative content exposure increases. The shaded areas represent approximate 95% confidence intervals.

#### Mediation analyses

Additional analyses examined whether psychological resilience mediated the relationships between the predictor variables and mental health outcomes [247, 248]. Results indicated significant indirect effects through resilience for both social media negative emotional content exposure (indirect effect = 0.12, *p* < 0.001, 95% CI [0.08, 0.16]) and mindful technology use (indirect effect = −0.19, *p* < 0.001, 95% CI [−0.24, −0.14]) [135, 249].

These mediation effects were partial rather than complete, as direct effects remained significant when including the mediator [250, 251]. This pattern suggests that resilience represents one important pathway through which emotional content exposure and mindful technology use influence mental health, but that additional mechanisms are also involved [99, 100, 252].

#### Effect sizes and practical significance

Effect sizes for the key findings were evaluated using established guidelines for SEM [253, 254]. Following conventional benchmarks (small ≈ 0.10, medium ≈ 0.30, large ≥ 0.50), the moderation of mindful technology use on emotional exposure represented a medium-to-large effect, explaining roughly 8 percent of additional variance in resilience. The moderation effect of mindful technology use (β = 0.32) indicates that for every one standard deviation increase in mindful technology use, resilience increased by approximately 0.32 standard deviations even under high emotional exposure. This effect represents a meaningful improvement in adaptive emotional functioning, highlighting the practical significance of mindful engagement in daily digital contexts. The secondary moderation by emotional contagion susceptibility added 3–4 percent incremental variance, consistent with prior digital psychology studies. The moderation effect of mindful technology use (β = 0.32) represented a medium to large effect size, indicating substantial practical significance [255, 256]. The main effect of mindful technology use on resilience (β = 0.45) represented a large effect size, suggesting that mindful technology use practices have meaningful real-world implications for psychological wellbeing [224, 257].

To provide additional context for the practical significance of these findings, we calculated the percentage of variance in resilience explained by the interaction terms [258, 259]. The mindful technology use moderation effect explained an additional 8.2% of variance in resilience beyond the main effects, while the digital emotional contagion susceptibility moderation explained an additional 3.7% of variance [135, 238].

### Supplementary analyses

#### Platform-specific effects

Exploratory analyses examined whether the observed effects varied across different social media platforms [260, 261]. Multi-group SEM analyses revealed that the moderation effects were strongest for Instagram users (β = 0.41, *p* < 0.001) and Facebook users (β = 0.35, *p* < 0.001), with smaller but still significant effects for Twitter/X users (β = 0.22, *p* < 0.05) [262, 263]. TikTok users showed non-significant moderation effects (β = 0.14, *p* = 0.12), though this may reflect the smaller subsample size for this platform [264, 265].

#### Demographic moderators

Additional analyses examined potential demographic moderators of the primary relationships [266, 267]. Age emerged as a significant moderator, with stronger protective effects of mindful technology use observed among older participants (ages 35 + years) compared to younger participants [268, 269]. Gender differences were not significant after controlling for other variables in the model [270, 271].

Educational level showed a marginal moderation effect (*p* = 0.067), with college-educated participants demonstrating somewhat stronger benefits from mindful technology use practices [272, 273]. These findings suggest that the Digital Emotional Regulation Paradox may be particularly relevant for certain demographic groups, though replication with larger samples is needed to confirm these patterns [274, 275].

## Discussion

The central aim of this study was to examine whether mindful technology use buffers the negative psychological effects of emotional content exposure on social media. The findings confirmed this moderating effect, demonstrating that mindful engagement transforms digital exposure from a source of stress into an opportunity for adaptive regulation and growth.

### Interpretation of primary findings

The results of this study provide strong support for the novel concept of the Digital Emotional Regulation Paradox, demonstrating that mindful technology use can serve as a significant protective factor against the negative psychological effects of emotional content exposure on social media. This paradox reflects that greater exposure to emotional content does not necessarily lead to psychological harm when individuals engage mindfully with technology.The finding that individuals with high levels of mindful technology use maintained psychological resilience even when exposed to negative emotional content challenges prevailing assumptions about digital content avoidance as the primary strategy for maintaining mental health in digital environments [81, 209]. Nevertheless, alternative explanations cannot be ruled out, as it is possible that individuals who are naturally more resilient also tend to engage more mindfully with technology. Future longitudinal and experimental studies could help disentangle this reciprocal relationship.

The moderation effect (β = 0.32) provides robust evidence that the quality of digital engagement, rather than its quantity, is the key determinant of resilience, indicating that individuals who approach technology use with awareness and intentionality can effectively regulate their emotional responses even under high emotional exposure [276]. The protective effects were most pronounced among individuals with low digital emotional contagion susceptibility, suggesting that mindful technology use practices may be particularly beneficial for those who are naturally less vulnerable to emotional influence from digital content [135, 277].

The three-way interaction findings reveal a nuanced picture of digital emotional regulation processes. For individuals with high emotional contagion susceptibility, mindful technology use provided partial but not complete protection against negative content effects. This suggests that vulnerable individuals may benefit from more intensive interventions that combine mindful technology use training with other emotion regulation strategies [99, 100, 278]. The complete buffering effect observed among low-susceptibility, high-mindful-use individuals provides compelling evidence for the potential of mindful technology practices to fundamentally alter the relationship between digital content exposure and psychological wellbeing [179, 273].

These findings highlight a shift from avoidance-based approaches toward engagement-based resilience strategies. While prior research has advocated digital detox or content avoidance as means to protect mental health [14], the current study demonstrates that mindful engagement can transform the emotional impact of social media exposure into opportunities for adaptive growth and self-regulation.

### Theoretical implications

These findings contribute to several important theoretical domains within digital psychology and emotion regulation research. First, they extend emotional contagion theory by identifying mindful awareness and intentional engagement as key moderators of contagion effects in digital environments [35, 279]. Traditional emotional contagion research has focused primarily on automatic, unconscious processes, but these results suggest that conscious regulatory strategies can significantly influence susceptibility to emotional influence [28, 280].

The study also advances understanding of self-determination theory in digital contexts by demonstrating that autonomous, values-based technology use promotes psychological wellbeing even in challenging digital environments [4, 281]. The strong main effects of mindful technology use on resilience and mental health outcomes support theoretical predictions that self-determined behavior promotes flourishing across diverse life domains [282, 283].

From a mindfulness perspective, these findings suggest that mindfulness principles can be successfully adapted to digital environments to promote emotional regulation [284, 285]. The concept of mindful technology use represents a novel application of mindfulness that maintains the core principles of present-moment awareness and values-based action while addressing the unique challenges of digital engagement [286, 287].

The present findings have direct implications for digital mental health interventions. Training programs that cultivate mindful awareness during online activity could help individuals recognize emotional triggers and respond with reflective rather than reactive engagement. Digital wellbeing curricula and app-based interventions might incorporate features such as mindful check-ins or guided pauses to support emotional regulation during online interactions [288].

These insights also suggest opportunities for clinicians and educators to integrate mindful technology use into stress management and resilience-building frameworks, particularly for adolescents and young adults navigating high emotional exposure in digital contexts [289, 290].

The results also have implications for resilience theory by identifying digital emotional regulation skills as a potentially important component of adaptive functioning in contemporary society [291]. As digital environments become increasingly central to social interaction and information consumption, the ability to navigate emotional content mindfully may represent a crucial 21st-century life skill [79, 288].

### Practical implications

#### Clinical applications

The findings have several important implications for clinical practice and digital mental health interventions. Mental health professionals working with clients who report distress related to social media use should consider assessing not only frequency of use but also quality of engagement patterns [292, 293]. The Mindful Technology Use Scale developed for this study could serve as a useful clinical assessment tool for identifying clients who might benefit from mindful technology use training [73, 294].

Intervention protocols could be developed that teach clients specific skills for engaging with emotional content on social media in ways that maintain or enhance psychological resilience [99, 100, 295]. These might include techniques for pausing before responding to emotional posts, setting intentional boundaries around exposure timing, and practicing awareness of emotional responses to digital content [296, 297].

The finding that mindful technology use benefits are strongest among individuals with low emotional contagion susceptibility suggests that interventions should be tailored based on individual vulnerability factors [135, 298]. Clients with high susceptibility may require more comprehensive approaches that address underlying emotional regulation skills in addition to technology-specific strategies [221, 299].

#### Educational applications

These findings have significant implications for digital literacy and wellbeing education programs in schools and universities. Current digital wellness curricula often emphasize reducing screen time and avoiding certain types of content, but these results suggest that teaching mindful engagement skills may be more effective [68, 300]. Educational programs could incorporate training in intentional technology use, emotional awareness during digital activities, and values-based decision-making about technology engagement [301, 302].

The age-related moderation effects observed in supplementary analyses suggest that mindful technology use interventions may be particularly beneficial for older adolescents and young adults who are developing their long-term technology habits [290, 303]. University counseling centers and student wellness programs could implement mindful technology use workshops as part of their mental health promotion efforts [289, 304].

#### Cultural context and socioreligious dimensions

The Indonesian cultural and spiritual context provides an important lens for interpreting the Digital Emotional Regulation Paradox. Indonesia’s collectivist values emphasize harmony, empathy, and communal responsibility, which may encourage mindful forms of online engagement that prioritize relational wellbeing over individual expression [305]. In such contexts, mindful technology use is not only a personal self-regulation practice but also a social ethic that reflects respect, balance, and empathy within online interactions.

Moreover, Indonesia’s strong religious traditions may influence how individuals integrate mindful technology use into their moral and spiritual frameworks. Practices such as reflective awareness, gratitude, and emotional restraint are often reinforced through spiritual teachings, providing a culturally embedded foundation for mindful engagement and emotional resilience. These socioreligious dimensions suggest that mindful technology use in collectivist societies may operate through mechanisms that blend psychological awareness with ethical and spiritual intentionality, distinguishing it from Western, individual-centered conceptualizations of mindful technology use.

#### Technology design implications

The results have important implications for the design of social media platforms and digital wellness tools. Technology companies could incorporate features that promote mindful engagement, such as prompts for users to pause and reflect before sharing emotional content or tools that help users track their emotional responses to different types of content [288, 306]. Platform algorithms could be designed to consider not just engagement metrics but also user wellbeing indicators when curating content [202, 307].

The finding that protective effects varied across platforms suggests that different social media environments may require different approaches to mindful engagement [308, 309]. Instagram and Facebook users showed the strongest benefits from mindful technology use, possibly reflecting the visual and personal nature of content on these platforms [310, 311]. Design features that support mindful use may be particularly important for platforms with high emotional content density [2, 312].

### Limitations and future research directions

Several limitations of this study should be acknowledged when interpreting the findings. Moreover, as this study was conducted within the Indonesian sociocultural context, patterns of emotional expression, technology use, and mindfulness practice may differ from those in Western populations. Cultural norms emphasizing community harmony and emotional restraint might influence both social media behavior and emotional regulation strategies, which limits the generalizability of the findings to other cultural settings. The cross-sectional design limits causal inferences about the relationships between mindful technology use and psychological outcomes [313, 314]. Future research should employ longitudinal or experimental designs to establish the directionality of these relationships and test whether mindful technology use training can improve resilience to digital emotional content [57, 315]. Future studies could employ longitudinal panel designs or randomized controlled experiments that examine how mindful technology use training affects emotional resilience over time. Experimental manipulations exposing participants to varying emotional content levels under mindful versus habitual use conditions could clarify the causal mechanisms underlying the Digital Emotional Regulation Paradox. These limitations may have influenced the observed relationships, potentially leading to underestimation or overestimation of certain effects. Future longitudinal and experimental studies are necessary to establish the directionality and causality of these associations.

The reliance on self-report measures introduces potential biases, including social desirability effects and common method variance [316, 317]. Future studies could incorporate objective measures of technology use behavior, physiological indicators of emotional responses, and behavioral observations to complement self-report data [318, 319]. The development of ecological momentary assessment protocols could provide more detailed and accurate information about real-time relationships between digital content exposure and emotional responses [320, 321]. To enhance ecological validity, future work could incorporate objective digital behavior tracking, such as usage logs, screen time analytics, and physiological data (e.g., heart rate variability), to complement self-reported experiences. These multimethod approaches would enable a richer understanding of real-time emotional regulation dynamics in digital contexts.

The sample consisted primarily of adults from Western, educated populations, which may limit generalizability to other cultural contexts and age groups [307, 322]. Cultural differences in emotion regulation strategies, technology use patterns, and social media norms could influence the effectiveness of mindful technology use approaches [2, 272]. Cross-cultural research is needed to examine whether the Digital Emotional Regulation Paradox generalizes across diverse populations and cultural contexts [47, 323].

The study focused specifically on social media platforms and did not examine other forms of digital emotional content exposure, such as news consumption, online gaming, or streaming media [324, 325]. Future research should investigate whether mindful technology use principles apply to these other digital contexts and whether different skills are needed for different types of digital environments [277, 326].

The measurement of emotional content exposure relied on participants’ subjective assessments rather than objective content analysis [327, 328]. Future studies could employ automated sentiment analysis of participants’ actual social media feeds to provide more objective measures of emotional content exposure [329, 330]. This approach would also allow for examination of specific types of emotional content (e.g., anger, sadness, anxiety) and their differential effects on psychological outcomes [56, 331]. Despite these limitations, the present study provides an important foundation for culturally grounded, empirically testable models of mindful digital engagement that can guide cross-national collaborations in digital mental health research.

Given that the sample consisted primarily of urban Indonesian adults, generalization to other cultural contexts should be approached with caution. Differences in cultural norms, emotional expression, and digital behavior across societies may influence how mindful technology use operates as a resilience mechanism. Replications in diverse cultural and age groups would strengthen the external validity of the Digital Emotional Regulation Paradox framework.

### Future research priorities

Several important directions for future research emerge from these findings. First, intervention studies are needed to test whether mindful technology use can be effectively taught and whether such training produces improvements in digital emotional regulation [2, 33]. Randomized controlled trials comparing mindful technology use training to other digital wellness interventions would provide important evidence for clinical and educational applications [332, 333].

Longitudinal research tracking individuals over time could examine how mindful technology use skills develop naturally and identify critical periods for intervention [334, 335]. Such studies could also investigate whether early exposure to mindful technology use principles influences long-term patterns of digital engagement and psychological wellbeing [224, 336].

Research examining the neurobiological mechanisms underlying mindful technology use effects could provide insights into how conscious regulatory strategies influence automatic emotional responses to digital content [337, 338]. Neuroimaging studies comparing brain activation patterns during mindful versus habitual technology use could illuminate the neural pathways involved in digital emotional regulation [339, 340].

The development and validation of objective measures of mindful technology use represents another important research priority [320, 341]. Behavioral indicators such as response latency to emotional content, patterns of content engagement, and physiological markers during technology use could provide convergent validity for self-report measures [342, 343].

Finally, research examining the boundary conditions and potential negative effects of mindful technology use approaches is needed to ensure that interventions are safe and appropriate for all individuals [16, 289]. Some individuals may experience increased anxiety or self-consciousness when attempting to engage mindfully with technology, and understanding these potential adverse effects is crucial for ethical intervention development [344, 345].

## Conclusion

This study provides compelling evidence for the Digital Emotional Regulation Paradox, demonstrating that mindful technology use can serve as a powerful protective factor against the negative psychological effects of emotional content exposure on social media. The findings challenge conventional wisdom about digital content avoidance and suggest that how individuals engage with technology—rather than how much they use it—may be the critical factor in determining psychological outcomes. The significant moderation effects observed indicate that individuals who approach social media with mindful awareness and intentional engagement can maintain psychological resilience even when exposed to negative emotional content.

The practical implications of these findings are substantial, offering new directions for clinical interventions, educational programs, and technology design practices. Rather than focusing solely on reducing screen time or avoiding certain types of content, mental health professionals and educators can help individuals develop sophisticated skills for navigating digital emotional landscapes. The concept of mindful technology use represents a promising framework for promoting digital wellbeing that builds on individual strengths and agency rather than imposing restrictions.

The theoretical contributions of this research extend our understanding of emotional contagion, self-determination, mindfulness, and resilience in digital contexts. By demonstrating that conscious regulatory strategies can significantly moderate automatic emotional responses to digital content, these findings advance theoretical models of emotion regulation and suggest new avenues for understanding human adaptation to technological environments.

While limitations exist and future research is needed to establish causal relationships and examine generalizability, the current findings provide a strong foundation for developing evidence-based approaches to digital mental health. As society continues to navigate the challenges and opportunities of digital connectivity, research on adaptive engagement strategies becomes increasingly critical for promoting psychological wellbeing across diverse populations and contexts.

The Digital Emotional Regulation Paradox represents a paradigm shift in thinking about digital wellness, moving from avoidance-based approaches to engagement-based strategies that empower individuals to thrive in digital environments. This research contributes to a growing understanding that technology can be a tool for enhancing rather than undermining psychological wellbeing when used with intention, awareness, and alignment with personal values. The implications extend beyond individual mental health to encompass broader questions about how society can harness the benefits of digital connectivity while mitigating potential risks.

Future research building on these findings has the potential to transform digital mental health interventions and contribute to the development of technology platforms that actively support human flourishing. By continuing to investigate the mechanisms and applications of mindful technology use, researchers can help individuals and communities develop the skills needed to navigate an increasingly connected world while maintaining psychological resilience and wellbeing.

## Supplementary Information

Supplementary Material 1

## Data Availability

The datasets generated and/or analyzed during the current study are available from the corresponding author on reasonable request.

## References

[1] Sangiorgio E, Di Marco N, Etta G, Cinelli M, Cerqueti R, Quattrociocchi W. Evaluating the effect of viral posts on social media engagement. Sci Rep. 2025;15(1):639. 10.1038/s41598-024-84960-6. 39753870 10.1038/s41598-024-84960-6 PMC11699135

[2] Verma A, Islam S, Moghaddam V, Anwar A. Digital emotion regulation on social media. Computer. 2024;57(6):82–9.

[3] Pecile G, Di Marco N, Cinelli M, Quattrociocchi W. Mapping the global election landscape on social media in 2024. PLoS One. 2025;20(2):e0316271. 39908218 10.1371/journal.pone.0316271 PMC11798462

[4] Peters H, Bayer JB, Matz SC, Chi Y, Vaid SS, Harari GM. Social media use is predictable from app sequences: using LSTM and transformer neural networks to model habitual behavior. Comput Human Behav. 2024;161:108381.

[5] Sabour S, Zhang W, Xiao X, Zhang Y, Zheng Y, Wen J, et al. A chatbot for mental health support: exploring the impact of Emohaa on reducing mental distress in China. Front Digit Health. 2023;5:1133987. 37214342 10.3389/fdgth.2023.1133987 PMC10193040

[6] Shan Y, Zhang J, Li Z, Feng Y, Zhou J. Mental health assessment for the Chatbots (No. arXiv:2201.05382). arXiv. 2022. 10.48550/arXiv.2201.05382.

[7] Dallimore KS, Sparks BA, Butcher K. The influence of angry customer outbursts on service providers’ facial displays and affective states. J Serv Res. 2007;10(1):78–92. 10.1177/1094670507304694.

[8] Guazzini A, Guidi E, Cecchini C, Milani M, Vilone D, Meringolo P. Self-presentation and emotional contagion on Facebook: New experimental measures of profiles’ emotional coherence (No. arXiv:1607.07243). arXiv. 2016. 10.48550/arXiv.1607.07243.

[9] Ludwig Vera U, Prieur L, Rennie SM, Beswerchij A, Weintraub D, Berry B, Wey J, Candido K, Platt ML. Synchronous smiles and hearts: dyadic meditations enhance closeness and prosocial behavior in virtual and in-person settings. Mindfulness2025. 10.1007/s12671-025-02588-7. 10.1007/s12671-025-02588-7 PMC12170796 40535578

[10] Ma Y, Zhang P, Xue L. Social contagion with emotional group interactions. Chaos Solitons Fractals. 2025;194:116143.

[11] Aleksandric A, Pankaj H, Wilson GM, Nilizadeh S. Sadness, anger, or anxiety: twitter users’ emotional responses to toxicity in public conversations (No. arXiv:2310.11436). arXiv. 2023. 10.48550/arXiv.2310.11436.

[12] Chandra Guntuku S, Preotiuc-Pietro D, Eichstaedt JC, Ungar LH. What Twitter profile and posted images reveal about depression and anxiety. Proc Int AAAI Conf Web Soc Media. 2019;13:236–46. 10.1609/icwsm.v13i01.3225.

[13] Lu T, Zheng H, Zhang T, Xu X “Orson,”, Guo A. InteractOut: Leveraging Interaction Proxies as Input Manipulation Strategies for Reducing Smartphone Overuse. Proceedings of the CHI Conference on Human Factors in Computing Systems. 2024. 1–19. 10.1145/3613904.3642317.

[14] Schmidt ME, Haines J, O’Brien A, McDonald J, Price S, Sherry B, et al. Systematic review of effective strategies for reducing screen time among young children. Obesity. 2012;20(7):1338–54. 10.1038/oby.2011.348. 22222926 10.1038/oby.2011.348

[15] Do QB, McKone KMP, Hamilton JL, Stone LB, Ladouceur CD, Silk JS. The link between adolescent girls’ interpersonal emotion regulation with parents and peers and depressive symptoms: a real-time investigation. Dev Psychopathol. 2025;37(1):1–15. 10.1017/S0954579423001359. 37933501 10.1017/S0954579423001359

[16] Lippold MA, McDaniel BT, Jensen TM. Mindful parenting and parent technology use: examining the intersections and outlining future research directions. Soc Sci. 2022;11(2):43.

[17] Kumar S, Jayant R, Charagulla N. Sentiment analysis on the news to improve mental health. 2021 IEEE MIT Undergraduate Research Technology Conference (URTC). 2021. 1–5. https://ieeexplore.ieee.org/abstract/document/9701632/.

[18] Pendse SR, Gergle D, Kornfield R, Meyerhoff J, Mohr D, Suh J, Wescott A, Williams C, Schleider J. When testing AI tests us: safeguarding mental health on the digital frontlines (no. arXiv:2504.20910). arXiv. 2025. 10.48550/arXiv.2504.20910. 10.1145/3715275.3732120 PMC13270898 42311791

[19] Avci H, Baams L, Kretschmer T. A systematic review of social media use and adolescent identity development. Adolesc Res Rev. 2025;10(2):219–36. 10.1007/s40894-024-00251-1. 40385471 10.1007/s40894-024-00251-1 PMC12084248

[20] Ayoub R. The commodity society. 2022. 10.5281/ZENODO.13322066.

[21] Champion A, Oswald F, Hughes S, Pedersen CL. Beyond just gender: diverse women’s experiences and outcomes associated with the receipt of unsolicited genital images. Sex Res Soc Policy. 2025;22(2):706–24. 10.1007/s13178-024-01002-6.

[22] Tselenti D, Cardoso D, Carvalho J. Framing empathy: examining audience responses to female-on-male sexual violence. Sex Cult. 2025;29(3):1491–512. 10.1007/s12119-025-10332-5.

[23] Liu J. “Lizzy,” Zhuo S, Li X, Dillon A, Howell N, Smith ADR, Zhang Y. From regulation to support: centering humans in technology-mediated emotion intervention in care contexts (No. arXiv:2504.12614). arXiv. 2025. 10.48550/arXiv.2504.12614.

[24] Kumar A, Kailasam AS, Rai A, Khanna M, Shukla S, Das S, Chakraborti A. The impact of meteorological factors on crop price volatility in India: case studies of Soybean and Brinjal (No. arXiv:2503.11690). arXiv. 2025. 10.48550/arXiv.2503.11690.

[25] Raugh IM, Strauss GP. Integrating mindfulness into the extended process model of emotion regulation: the dual-mode model of mindful emotion regulation. Emotion. 2024;24(3):847–66. 10.1037/emo0001308. 37843512 10.1037/emo0001308 PMC11009092

[26] Faghihi E, Zarenejad M, Shirazi AAB. SPECTRUM: semantic processing and emotion-informed video-captioning through retrieval and understanding modalities (No. arXiv:2411.01975). arXiv. 2024. 10.48550/arXiv.2411.01975.

[27] Mittal T, Mathur P, Chandra R, Bhatt A, Gupta V, Mukherjee D, Bera A, Manocha D. Estimating emotion contagion on social media via localized diffusion in dynamic graphs (No. arXiv:2207.07165). arXiv. 2022. 10.48550/arXiv.2207.07165.

[28] Khrennikov A. Quantum-like model for unconscious–conscious interaction and emotional coloring of perceptions and other conscious experiences. Biosystems. 2021;208:104471. 34237350 10.1016/j.biosystems.2021.104471

[29] Sonnby-Borgström M, Jönsson P, Svensson O. Imitative responses and verbally reported emotional contagion from spontaneous, unconscious to emotionally regulated, conscious information-processing levels. Neuropsychoanalysis. 2008;10(1):81–98. 10.1080/15294145.2008.10773573.

[30] Gertz N. Autonomy online: Jacques Ellul and the Facebook emotional manipulation study. Res Ethics. 2016;12(1):55–61. 10.1177/1747016115579534.

[31] Kramer ADI, Guillory JE, Hancock JT. Experimental evidence of massive-scale emotional contagion through social networks. Proc Natl Acad Sci U S A. 2014;111(24):8788–90. 10.1073/pnas.1320040111. 24889601 10.1073/pnas.1320040111 PMC4066473

[32] Southerton C, Taylor E. Habitual disclosure: routine, affordance, and the ethics of young peoples social media data surveillance. Soc Media Soc. 2020;6(2):2056305120915612. 10.1177/2056305120915612.

[33] Suzuki HN, Inaba M. Digital nudges using emotion regulation to reduce online disinformation sharing (No. arXiv:2503.24037). arXiv. 2025. 10.48550/arXiv.2503.24037.

[34] Fan R, Xu K, Zhao J. An agent-based model for emotion contagion and competition in online social media. Physica A. 2018;495:245–59. 10.1016/j.physa.2017.12.086.

[35] Ohara R, Yang CL, Narumi T, Kuzuoka H. Understanding and supporting co-viewing comedy in VR with embodied expressive avatars (No. arXiv:2505.20082). arXiv. 2025. 10.48550/arXiv.2505.20082.

[36] Eisenberger NI, Lieberman MD, Williams KD. Does rejection hurt? An fMRI study of social exclusion. Science. 2003;302(5643):290–2. 10.1126/science.1089134. 14551436 10.1126/science.1089134

[37] Zotev V, Phillips R, Yuan H, Misaki M, Bodurka J. Self-regulation of human brain activity using simultaneous real-time fMRI and EEG neurofeedback. Neuroimage. 2014;85:985–95. 23668969 10.1016/j.neuroimage.2013.04.126

[38] Jola C, Abedian-Amiri A, Kuppuswamy A, Pollick FE, Grosbras M-H. Motor simulation without motor expertise: enhanced corticospinal excitability in visually experienced dance spectators. PLoS One. 2012;7(3):e33343. 22457754 10.1371/journal.pone.0033343 PMC3310063

[39] Kumar H, Li T, Shi J, Musabirov I, Kornfield R, Meyerhoff J, et al. Using adaptive bandit experiments to increase and investigate engagement in mental health. Proc AAAI Conf Artif Intell. 2024;38(21):22906–12. 38666291 10.1609/aaai.v38i21.30328 PMC11044947

[40] Schierenbeck S, Rette D, Reyes-Portillo J. Impact of negative social media experiences on depression, anxiety, and suicidality among college students: exploring racial/ethnic differences. TMS Proceedings. 2021;2021. https://assets.pubpub.org/6i7jkggp/21635176789704.pdf.

[41] Bagherian M, Azadi M, Abdullahpour MA. The effectiveness of group psychodrama on life satisfaction, self-compassion, and positive emotions in depressed patients. J Psychol Dynamics Mood Disord. 2024;3(2):86–96. 10.61838/kman.pdmd.3.2.8.

[42] Baranik LE, Eby L. Organizational citizenship behaviors and employee depressed mood, burnout, and satisfaction with health and life: the mediating role of positive affect. Personnel Rev. 2016;45(4):626–42.

[43] Alessandri G, Filosa L, Sonnentag S, Crea G, Borgogni L, Avanzi L, et al. Determinants of workers’ well-being during the COVID-19 outbreak: an exploratory study. Curr Psychol. 2023;42(10):8595–614. 10.1007/s12144-021-02408-w. 34703195 10.1007/s12144-021-02408-w PMC8531915

[44] Suls J, Green P, Hillis S. Emotional reactivity to everyday problems, affective inertia, and neuroticism. Pers Soc Psychol Bull. 1998;24(2):127–36. 10.1177/0146167298242002.

[45] Das Swain V, Zhong Q. “Joy,” Parekh JR, Jeon Y, Zimmermann R, Czerwinski MP, Suh J, Mishra V, Saha K, Hernandez J. AI on my shoulder: supporting emotional labor in front-office roles with an llm-based empathetic coworker. Proceedings of the 2025 CHI Conference on Human Factors in Computing Systems. 2025. 1–29. 10.1145/3706598.3713705.

[46] Ma C, Xu Z, Ren Y, Hettiachchi D, Chan J. PUB: an LLM-enhanced personality-driven user behaviour simulator for recommender system evaluation. 2025. arXiv Preprint arXiv:2506.04551. https://arxiv.org/abs/2506.04551.

[47] Ma Y, Zhang Y, Fu D, Zubicueta Portales S, Kragic D, Fjeld M. Advancing user-voice interaction: exploring emotion-aware voice assistants through a role-swapping approach. In N. A. Streitz & S. Konomi (Eds.), Distributed, Ambient and Pervasive Interactions (Vol. 15802). Springer Nature Switzerland. 2025. pp. 303–320. 10.1007/978-3-031-92977-9_19.

[48] Maraule M, Duffett R, Edu T. Modeling emoji online marketing on websites among young consumers: the moderation effect of age. Future Bus J. 2025;11(1):91. 10.1186/s43093-025-00509-7.

[49] Oleszkiewicz A, Karwowski M, Pisanski K, Sorokowski P, Sobrado B, Sorokowska A. Who uses emoticons? Data from 86 702 Facebook users. Pers Individ Differ. 2017;119:289–95.

[50] Marshall B, Warburton WA, Kangas M, Sweller N. Internet gaming disorder (IGD) and smartphone overuse in Australian primary school and secondary school children: prevalence and developmental impacts. Curr Psychol2025. 10.1007/s12144-025-07975-w.

[51] Macedo M, Saxena A. Gender differences in online communication: a case study of Soccer (No. arXiv:2403.11051). arXiv. 2024. 10.48550/arXiv.2403.11051.

[52] Truong V. Examining gender and cultural influences on customer emotions (No. arXiv:2505.02852). arXiv. 2025. 10.48550/arXiv.2505.02852.

[53] Guan W. The social contagion of adolescent depression: applying a differential susceptibility model. Louisiana State University and Agricultural & Mechanical College. 2016. https://search.proquest.com/openview/51070b804522db6d2db5ae8260e0270c/1?pq-origsite=gscholar%26cbl=18750%26diss=y.

[54] Guntuku SC, Preotiuc-Pietro D, Eichstaedt JC, Ungar LH. What twitter profile and posted images reveal about depression and anxiety. Proceedings of the International AAAI Conference on Web and Social Media. 2019;13:236–246. https://aaai.org/ojs/index.php/ICWSM/article/view/3225.

[55] Bayer JB, Ellison NB, Schoenebeck SY, Falk EB. Sharing the small moments: ephemeral social interaction on Snapchat. Inf Commun Soc. 2016;19(7):956–77. 10.1080/1369118X.2015.1084349.

[56] Yu Y, Huang S, Liu Y, Tan Y. Emotions in online content diffusion. 2020. arXiv Preprint arXiv:2011.09003. https://arxiv.org/abs/2011.09003.

[57] Mendu S, Doyle Fosco SL, Lanza ST, Abdullah S. Designing voice interfaces to support mindfulness-based pain management. Digit Health. 2023;9:20552076231204418. 10.1177/20552076231204418. 37868159 10.1177/20552076231204418 PMC10588404

[58] Syed M, Byrne L, Lum J, Skvarc D. A survey of engagement with mindfulness and brain training apps. J Cogn Enhancement. 2025.10.1007/s41465-025-00324-6.

[59] Fullerton K. We need to understand it before we can teach it: A cooperative inquiry into mindfulness-based social-emotional learning with international school teachers [PhD Thesis, Antioch University]. 2024. https://search.proquest.com/openview/a14af824c076429ef3cc01cd226df1b8/1?pq-origsite=gscholar&cbl=18750&diss=y.

[60] Rahmani Z, Shahini N, Gat N, Yun Z, Jiang Y, Farchy O, Harel Y, Chaudhary V, Ayday E, Sharif M. Privacy-preserving collaborative genomic research: a real-life deployment and vision. Proceedings of the 2024 Workshop on Cybersecurity in Healthcare. 2023. 85–91. 10.1145/3689942.3694747.

[61] Adanyin A. AI-driven feedback loops in digital technologies: psychological impacts on user behaviour and well-being (No. arXiv:2411.09706). arXiv. 2024. 10.48550/arXiv.2411.09706.

[62] Diamond RV. User experience, software interfaces, and the unconscious (No. arXiv:0909.1138). arXiv. 2009. 10.48550/arXiv.0909.1138.

[63] Svikhnushina E, Pu P. Social and emotional etiquette of chatbots: a qualitative approach to understanding user needs and expectations (No. arXiv:2006.13883). arXiv. 2020. 10.48550/arXiv.2006.13883.

[64] Zhang Y, Stewart C, Ranjan Y, Conde P, Sankesara H, Rashid Z, Sun S, Dobson RJ, Folarin AA. Large-scale digital phenotyping: Identifying depression and anxiety indicators in a general UK population with over 10,000 participants. J Affect Disord. 2025;375:412–422. 10.1016/j.jad.2025.01.124 39892753

[65] Gyrard A, Mohammadi S, Gaur M, Kung A. IoT-based preventive mental health using knowledge graphs and standards for better well-being (No. arXiv:2406.13791). arXiv. 2024. 10.48550/arXiv.2406.13791.

[66] Walsh LC, Regan A, Okabe-Miyamoto K, Lyubomirsky S. Does putting down your smartphone make you happier? The effects of restricting digital media on well-being. PLoS One. 2024;19(10):e0306910. 39401227 10.1371/journal.pone.0306910 PMC11472914

[67] Salem MB. Overcoming inner and outer constraints to enhance emerging adult college students’ eudaemonic well-being: a mixed methods study [PhD Thesis, University of Northern Colorado]. 2022. https://search.proquest.com/openview/619318ddbd341644fa1aba503dc66243/1.pdf?pq-origsite=gscholar&cbl=18750&diss=y.

[68] Zhao Y, Li T, Sobolev M. Digital wellbeing redefined: toward user-centric approach for positive social media engagement. Proceedings of the IEEE/ACM 11th International Conference on Mobile Software Engineering and Systems. 2024. 95–98. 10.1145/3647632.3651392.

[69] Karanika-Murray M, Pontes HM, Griffiths MD, Biron C. Sickness presenteeism determines job satisfaction via affective-motivational states. Soc Sci Med. 2015;139:100–6. 10.1016/j.socscimed.2015.06.035. 26183017 10.1016/j.socscimed.2015.06.035

[70] Tessier D, Sarrazin P, Ntoumanis N. The effect of an intervention to improve newly qualified teachers’ interpersonal style, students motivation and psychological need satisfaction in sport-based physical education. Contemp Educ Psychol. 2010;35(4):242–53.

[71] Behaein N, Farsi A, Franz E, Lipowski M. Effects of biofeedback and mindfulness on the psychological skills and forehand flick accuracy in adolescent table tennis players. J Rational-Emot Cogn Behav Ther. 2025;43(1):17. 10.1007/s10942-025-00581-6.

[72] Weisel KK, Fuhrmann LM, Berking M, Baumeister H, Cuijpers P, Ebert DD. Standalone smartphone apps for mental health—a systematic review and meta-analysis. NPJ Digit Med. 2019;2(1):118. 31815193 10.1038/s41746-019-0188-8 PMC6889400

[73] Clawson B. Systematic review regarding the use of mindfulness-based mobile applications to reduce psychological symptoms and enhance well-being among general and clinical population adults: benefits, limitations and future directions. Pepperdine University. 2022. https://search.proquest.com/openview/353a64931e5dead30ccb9a53de454002/1?pq-origsite=gscholar&cbl=18750&diss=y.

[74] Jeong S, Alghowinem S, Aymerich-Franch L, Arias K, Lapedriza A, Picard R, Park HW, Breazeal C. A robotic positive psychology coach to improve college students’ wellbeing. 2020 29th IEEE International Conference on Robot and Human Interactive Communication (RO-MAN). 2020;187–194. https://ieeexplore.ieee.org/abstract/document/9223588/.

[75] Alhassan MD, Butler M. Digital resilience and the continuance use of mobile payment services. 2021.

[76] Okuyama J, Seto S, Motokawa T, Kato T, Miyamoto A, Maekawa M, et al. Digital support for female students in physical education universities in Japan. Sci Rep. 2025;15(1):1–16. 40369037 10.1038/s41598-025-98921-0 PMC12078689

[77] Herrera LC. Resilience through crisis management transitions: a process organization study of support information systems in public service, the case of social media analytics. Doctoral Dissertations at University of Agder. 2025.

[78] Ribeiro C. Unlocking banking’s digital future: santander digital transformation odyssey. 2024. https://www.proquest.com/openview/0d8178c284cd394565d4c22a9914658d/1?pq-origsite=gscholar&cbl=2026366&diss=y.

[79] Laban G, Morrison V, Kappas A, S. Cross, E. Coping with emotional distress via self-disclosure to robots: an intervention with caregivers. Int J Soc Robotics. 2025. 10.1007/s12369-024-01207-0. 10.1007/s12369-024-01207-0 PMC12460501 41020173

[80] Lin L. Can you get emotional support through a screen? A look into digital and in-person emotional support and emotion regulation. Inquiry@ Queen’s Undergraduate Research Conference Proceedings. 2019. https://ojs.library.queensu.ca/index.php/inquiryatqueens/article/view/13383.

[81] Kulkarni M, Durve S, Jia B. Cyberbully and online harassment: issues associated with digital wellbeing (No. arXiv:2404.18989). arXiv. 2024. 10.48550/arXiv.2404.18989.

[82] Trimpey J. An exploration of social networking use and mental health in transgender, gender non-conforming, gender non-binary, and gender fluid persons. 2020. https://repository.usfca.edu/diss/525/.

[83] Niu C. Impact of missing data and ICC on full information maximum-likelihood estimation in multilevel SEMs. Model Assist Stat Appl. 2024;19(1):49–59. 10.3233/MAS-231444.

[84] Hammond SP, Polizzi G, Duddy C, Bennett-Grant Y, Bartholomew KJ. Children’s, parents’ and educators’ understandings and experiences of digital resilience: a systematic review and meta-ethnography. New Media Soc. 2024;26(5):3018–42. 10.1177/14614448241232065.

[85] Lyons M, Bootes E, Brewer G, Stratton K, Centifanti L. COVID-19 spreads round the planet, and so do paranoid thoughts”. A qualitative investigation into personal experiences of psychosis during the COVID-19 pandemic. Curr Psychol. 2023;42(13):10826–35. 10.1007/s12144-021-02369-0. 34658609 10.1007/s12144-021-02369-0 PMC8505012

[86] Yang Q, Feng S, Zhao T, Kalantari S. Co-design with myself: a brain-computer interface design tool that predicts live emotion to enhance metacognitive monitoring of designers. Extended Abstracts of the 2023 CHI Conference on Human Factors in Computing Systems. 2023. 1–8. 10.1145/3544549.3585701.

[87] Acheampong R, Balan TC, Popovici D-M, Tuyishime E, Rekeraho A, Voinea GD. Balancing usability, user experience, security and privacy in XR systems: a multidimensional approach. Int J Inf Secur. 2025;24(3):112. 10.1007/s10207-025-01025-z.

[88] Kang S. Toward a healthier social media experience: Designing’Inspiration’and’Reality’Modes to Enhance Digital Well-Being for Generation Z. 2025. arXiv Preprint arXiv:2503.21195. https://arxiv.org/abs/2503.21195.

[89] Huang J, Amey RC, Liu M, Forbes CE. Functional graph contrastive learning of hyperscanning EEG reveals emotional contagion evoked by stereotype-based stressors. Front Hum Neurosci. 2023;17:1298845. 38077186 10.3389/fnhum.2023.1298845 PMC10698865

[90] Sharp J, Kelson J, South D, Saliba A, Kabir MA. Virtual reality and artificial intelligence as psychological countermeasures in space and other isolated and confined environments: a scoping review. Acta Astronautica. 2025. https://www.sciencedirect.com/science/article/pii/S0094576525002024.

[91] Hors-Fraile S, Rivera-Romero O, Schneider F, Fernandez-Luque L, Luna-Perejon F, Civit-Balcells A, et al. Analyzing recommender systems for health promotion using a multidisciplinary taxonomy: a scoping review. Int J Med Inform. 2018;114:143–55. 10.1016/j.ijmedinf.2017.12.018. 29331276 10.1016/j.ijmedinf.2017.12.018

[92] Wang D, Li Q, Chaves Lima L, Grue Simonsen J, Lioma C. Contextual compositionality detection with external knowledge bases and word embeddings. Companion Proceedings of The 2019 World Wide Web Conference. 2019. 317–323. 10.1145/3308560.3316584.

[93] Klein Y, Nilsen IBR, Lindfors P, Hanson LLM, Stenfors CUD. Nature visits buffered against loneliness during COVID-19, especially among those mainly working remotely: a population-based study of working adults in Sweden. J Public Health.202510.1007/s10389-025-02465-6

[94] Morini V, Citraro S, Sajno E, Sansoni M, Riva G, Stella M, Rossetti G. Online posting effects: Unveiling the non-linear journeys of users in depression communities on Reddit (No. arXiv:2311.17684). arXiv. 2025. 10.48550/arXiv.2311.17684.

[95] Banyard V, Hamby S, Grych J. Health effects of adverse childhood events: identifying promising protective factors at the intersection of mental and physical well-being. Child Abuse Negl. 2017;65:88–98. 28131000 10.1016/j.chiabu.2017.01.011

[96] Pan Q, Lan M, Tan CY, Tao S, Liang Q, Law N. Protective factors contributing to adolescents’ multifaceted digital resilience for their wellbeing: a socio-ecological perspective. Comput Human Behav. 2024;155:108164.

[97] Duan S, Wang Z, Wang S, Chen M, Zhang R. Emotion-aware interaction design in intelligent user interface using multi-modal deep learning. 2024 5th International Symposium on Computer Engineering and Intelligent Communications (ISCEIC). 2024. 110–114. 10.1109/ISCEIC63613.2024.10810240.

[98] Slipetz LR, Qiu J, Sun S, Henry TR. Identifying nonlinear relations among random variables: a network analytic approach (No. arXiv:2411.02763). arXiv. 2024. 10.48550/arXiv.2411.02763.

[99] Wang W, Mensah IA, Atingabili S, Omari-Sasu AY. Climate change as a game changer: rethinking Africa’s food security-health outcome nexus through a multi-sectoral lens. Sci Rep. 2025;15(1):1–25. 40369173 10.1038/s41598-025-99276-2 PMC12078603

[100] Wang X, Larson MG, Liu C. Two-stage multiple imputation with a longitudinal composite variable. BMC Med Res Methodol. 2025;25(1):124. 10.1186/s12874-025-02555-9. 40329169 10.1186/s12874-025-02555-9 PMC12054270

[101] Brauer K, Sendatzki R, Proyer RT. A primer on studying effects of relationship duration in dyadic research: contrasting cross-sectional and longitudinal approaches. J Soc Pers Relat. 2022;39(7):2117–33. 10.1177/02654075221074677.

[102] Ignatavičienė K, Žukauskienė R. The possibility to combine longitudinal and cross-sectional design for studying development. Psichologija. 2002;25:64–71.

[103] Al Koliby IS, Noor NHM, Al-Swidi AK, Al-Hakimi MA, Mehat NAB. Enhancing sustainable performance among manufacturing SMEs: the interplay of knowledge management and organizational structure. Discover Sustain. 2025;6(1):469. 10.1007/s43621-025-01351-1.

[104] Oriol Granado X, Miranda Ayala R, Varela J, Garcia-Blanc N. Bullying victimization and subjective well-being in 10-and 12-year-old children from 24 Countries: the buffering effect of family and teacher support. 2025. https://recercat.cat/handle/10256/26561.

[105] Shani C, Stade EC. Measuring mental health variables in computational research: toward validated, dimensional, and transdiagnostic approaches (No. arXiv:2504.13890). arXiv. 2025. 10.48550/arXiv.2504.13890.

[106] Yang Q, Wang Z, Chen H, Wang S, Pu Y, Gao X, Huang W, Song S, Huang G. PsychoGAT: a novel psychological measurement paradigm through interactive fiction games with LLM agents (No. arXiv:2402.12326). arXiv. 2024. 10.48550/arXiv.2402.12326.

[107] Shmueli B, Fell J, Ray S, Ku LW. Beyond Fair Pay: Ethical Implications of NLP Crowdsourcing. Proceedings of the 2021 Conference of the North American Chapter of the Association for Computational Linguistics: Human Language Technologies. 2021. 3758–3769. 10.18653/v1/2021.naacl-main.295.

[108] Sikeridis D, Papapanagiotou I, Devetsikiotis M. BLEBeacon: A real-subject trial dataset from mobile bluetooth low energy beacons (No. arXiv:1802.08782). arXiv. 2019. 10.48550/arXiv.1802.08782.

[109] Hunter K, Miratrix L, Porter K. Power Under Multiplicity Project (PUMP): estimating power, minimum detectable effect size, and sample size when adjusting for multiple outcomes in multi-level experiments (No. arXiv:2112.15273). arXiv. 2023. 10.48550/arXiv.2112.15273.

[110] Lin J, Qian T. Micro-randomized trials with categorical treatments: causal effect estimation and sample size calculation (No. arXiv:2504.15484). arXiv. 2025. 10.48550/arXiv.2504.15484.

[111] Lora SK, Purba SA, Hossain B, Oriana T, Seum A, Sharmin S. Infinite Scrolling, Finite Satisfaction: Exploring User Behavior and Satisfaction on Social Media in Bangladesh (No. arXiv:2408.09601). arXiv. 2025. 10.48550/arXiv.2408.09601.

[112] Lee H, Paul U, Gupta A, Belding E, Gu M. Analyzing Disparity and Temporal Progression of Internet Quality through Crowdsourced Measurements with Bias-Correction (No. arXiv:2310.16136). arXiv. 2023. 10.48550/arXiv.2310.16136.

[113] Islam ZU. The role of social media in enhancing English language proficiency. J Language Linguistics Soc. 2022;24:54–64.

[114] Pham N, Pham L, Meyers AL. Towards better inclusivity: a diverse tweet corpus of english varieties (No. arXiv:2401.11487). arXiv. 2024. 10.48550/arXiv.2401.11487.

[115] Hagyari-Donaldson P, Scott N. Online therapy for children: yay or nay? Clinicians’ insights from the COVID-19 era. Child Youth Care Forum. 2025;54(3):687–714. 10.1007/s10566-024-09835-3.

[116] Mair JL, Salamanca-Sanabria A, Augsburger M, Frese BF, Abend S, Jakob R, et al. Effective behavior change techniques in digital health interventions for the prevention or management of noncommunicable diseases: an umbrella review. Ann Behav Med. 2023;57(10):817–35. 37625030 10.1093/abm/kaad041 PMC10498822

[117] Ge Z, Hu N, Li D, Wang Y, Qi S, Xu Y, Shi H, Zhang J. A survey of large language models in mental health disorder detection on social media (No. arXiv:2504.02800). arXiv. 2025. 10.48550/arXiv.2504.02800.

[118] Rahmadiana M, Karyotaki E, Schulte M, Ebert DD, Passchier J, Cuijpers P, et al. Transdiagnostic internet intervention for Indonesian university students with depression and anxiety: evaluation of feasibility and acceptability. JMIR Ment Health. 2021;8(3):e20036. 33666553 10.2196/20036 PMC7980121

[119] Faklaris C, Lipford HR, Tabassum S. Preliminary Results from a U.S. Demographic analysis of SMiSh susceptibility (No. arXiv:2309.06322). arXiv. 2023. 10.48550/arXiv.2309.06322.

[120] Pacheco E. The social media use of adult New Zealanders: evidence from an online survey (No. arXiv:2305.00119). arXiv. 2023. 10.48550/arXiv.2305.00119.

[121] Xiao Y, Du J, Zhang S, Zhang W, Yan Q, Zhang D, Kifer D. Click without compromise: online advertising measurement via per user differential privacy (No. arXiv:2406.02463). arXiv. 2025. 10.48550/arXiv.2406.02463.

[122] Guinalíu M, Díaz De Rada V. Combining sources of information to increase survey response rates. Spanish J Market. 2021;25(1):29–45. 10.1108/SJME-04-2020-0060.

[123] Paranjape CS, De Araujo OB, Reider LM, Sponseller PD, Carlini AR, McLaughlin K, et al. Time to completion of pediatric PROMIS computerized adaptive testing measures and the SRS-22r in an adolescent idiopathic scoliosis population. J Pediatr Orthop. 2022;42(9):462–6. 35973055 10.1097/BPO.0000000000002245 PMC9474712

[124] Wen S, Middleton M, Ping S, Chawla NN, Wu G, Feest BS, Nadri C, Liu Y, Kaber D, Zahabi M. AdaptiveCoPilot: design and testing of a neuroadaptive LLM cockpit guidance system in both novice and expert pilots. 2025 IEEE Conference Virtual Reality and 3D User Interfaces (VR). 2025. 656–666. https://ieeexplore.ieee.org/abstract/document/10937388/.

[125] Edwards SJL. Research participation and the right to withdraw. Bioethics. 2005;19(2):112–30. 10.1111/j.1467-8519.2005.00429.x. 15943021 10.1111/j.1467-8519.2005.00429.x

[126] Grant NK, Hamilton LK, Ormita JM. Improving comprehension of consent forms in online research: an empirical test of four interventions. J Empir Res Hum Res Ethics. 2025;20(1–2):46–54. 10.1177/15562646251321132. 40084819 10.1177/15562646251321132 PMC12048739

[127] Brown KK, Thomas SP, Brothers RM, Liao Y. “Lord Knows What’s Being Done with My Blood!”: Black Women’s perceptions of biospecimen donation for clinical research in the United States. J Racial Ethn Health Disparities. 2025;12(3):1856–65. 10.1007/s40615-024-02015-y. 38714639 10.1007/s40615-024-02015-y

[128] Gommesen NJ. Towards democratic data agency: attitudes and concerns about online data practices (No. arXiv:2503.05058). arXiv. 2025. 10.48550/arXiv.2503.05058.

[129] Schell C, Godinho A, Cunningham JA. Using a consistency check during data collection to identify invalid responding in an online cannabis screening survey. BMC Med Res Methodol. 2022;22(1):67. 10.1186/s12874-022-01556-2. 35282830 10.1186/s12874-022-01556-2 PMC8918323

[130] Welz M, Alfons A. When respondents don’t care anymore: identifying the onset of careless responding (No. arXiv:2303.07167). arXiv. 2025. 10.48550/arXiv.2303.07167.

[131] Canessa S, Martel A, Pasmans F. Designing screening protocols for amphibian disease that account for imperfect and variable capture rates of individuals. Ecol Appl. 2014;24(5):1204–12. 10.1890/13-0103.1. 25154107 10.1890/13-0103.1

[132] Severin KP. Spatial and temporal variation of *Marginopora vertebralis* on seagrass in Papua New Guinea during a six week period. Micropaleontology. 1987. 10.2307/1485574.

[133] Markowitz DM. From complexity to clarity: how AI enhances perceptions of scientists and the public’s understanding of science. PNAS Nexus. 2024;3(9):pgae387. 39290437 10.1093/pnasnexus/pgae387 PMC11406778

[134] Rasuli B. Publish for public: improving access of public libraries users to research findings through plain language summaries (No. arXiv:2307.16192). arXiv. 2023. 10.48550/arXiv.2307.16192.

[135] Ferrara E, Yang Z. Measuring emotional contagion in social media. PLoS One. 2015;10(11):e0142390. 10.1371/journal.pone.0142390. 26544688 10.1371/journal.pone.0142390 PMC4636231

[136] Henry TR, Slipetz LR, Falk A, Qiu J, Chen M. Ordinal outcome state-space models for intensive longitudinal data. Psychometrika. 2024;89(4):1203–29. 10.1007/s11336-024-09984-3. 38861220 10.1007/s11336-024-09984-3 PMC11582181

[137] Jeckeln G, Yavuzcan S, Marquis KA, Mehta PS, Yates AN, Phillips PJ, O’Toole AJ. Human-machine comparison for cross-race face verification: race bias at the upper limits of performance? (No. arXiv:2305.16443). arXiv. 2023. 10.48550/arXiv.2305.16443.

[138] Dowthwaite L, Vallejos EP, Creswick H, Portillo V, Patel M, Zhao J. Developing a measure of online wellbeing and user trust (No. arXiv:2007.14273). arXiv. 2020. 10.48550/arXiv.2007.14273.

[139] Graziotin D, Lenberg P, Feldt R, Wagner S. Psychometrics in behavioral software engineering: a methodological introduction with guidelines. ACM Trans Softw Eng Methodol. 2022;31(1):1–36. 10.1145/3469888.

[140] Lyroni C, Spais G. Consumer (brand) happiness of premium fashion brands and value consciousness: the case of clothing and footwear for Gen X and Gen Z as shoppers via digital platforms. J Market Anal.10.1057/s41270-024-00342-x.

[141] Park T, Reilly-Spong M, Gross CR. Mindfulness: a systematic review of instruments to measure an emergent patient-reported outcome (PRO). Qual Life Res. 2013;22(10):2639–59. 10.1007/s11136-013-0395-8. 23539467 10.1007/s11136-013-0395-8 PMC3745812

[142] Azcona EA, Kim B-W, Vike NL, Bari S, Lalvani S, Stefanopoulos L, et al. Discrete, recurrent, and scalable patterns in human judgement underlie affective picture ratings. Cogn Process. 2025;26(2):257–81. 10.1007/s10339-024-01250-9. 39644430 10.1007/s10339-024-01250-9 PMC12055920

[143] Brown KW, Ryan RM. The benefits of being present: mindfulness and its role in psychological well-being. J Pers Soc Psychol. 2003;84(4):822. 12703651 10.1037/0022-3514.84.4.822

[144] Lau MA, Bishop SR, Segal ZV, Buis T, Anderson ND, Carlson L, et al. The toronto mindfulness scale: development and validation. J Clin Psychol. 2006;62(12):1445–67. 10.1002/jclp.20326. 17019673 10.1002/jclp.20326

[145] Carreno DF, Eisenbeck N, Greville J, Wong PTP. Cross-cultural psychometric analysis of the mature happiness scale-revised: mature happiness, psychological inflexibility, and the PERMA model. J Happiness Stud. 2023;24(3):1075–99. 10.1007/s10902-023-00633-7. 36820217 10.1007/s10902-023-00633-7 PMC9932412

[146] Connor KM, Davidson JRT. Development of a new resilience scale: the Connor-Davidson Resilience Scale (CD-RISC). Depress Anxiety. 2003;18(2):76–82. 10.1002/da.10113. 12964174 10.1002/da.10113

[147] Skaldere-Darmudasa G, Sudraba V. Connor-Davidson resilience scale (CD-RISC-25) adaptation in Latvian sample. Soc Integration Educ Proc Int Sci Conf. 2023;2:488–97.

[148] Minnett K, Stephenson Z. Exploring the psychometric properties of the Connor-Davidson Resilience Scale (CD-RISC). Adversity Resilience Sci. 2024.10.1007/s42844-024-00159-8.

[149] Ryan RM, Frederick C. On energy, personality, and health: subjective vitality as a dynamic reflection of well-being. J Pers. 1997;65(3):529–65. 10.1111/j.1467-6494.1997.tb00326.x. 9327588 10.1111/j.1467-6494.1997.tb00326.x

[150] Prikhidko A, Long H, Wheaton MG. The effect of concerns about COVID-19 on anxiety, stress, parental burnout, and emotion regulation: the role of susceptibility to digital emotion contagion. Front Public Health. 2020;8:567250. 33392126 10.3389/fpubh.2020.567250 PMC7775569

[151] Tan S, Ji B, Pan Y. Style2talker: high-resolution talking head generation with emotion style and art style. Proc AAAI Conf Artif Intell. 2024;38(5):5079–87.

[152] Marx AK, Frenzel AC, Fiedler D, Reck C. Susceptibility to positive versus negative emotional contagion: first evidence on their distinction using a balanced self-report measure. PLoS One. 2024;19(5):e0302890. 38743712 10.1371/journal.pone.0302890 PMC11093349

[153] AK U, AO M, SK M, LE M, LA P. Validation of the kazakh version of the depression anxiety stress scale (Dass-21) In Medical Faculty Staff Sample: The Pilot Study: Baлидизaция Кaзaxcкoй Bepcии Oпpocникa Depression Anxiety Stress Scale (Dass-21) Cpeди Пpoфeccopcкo-Пpeпoдaвaтeльcкoгo Cocтaвa Meдицинcкиx Унивepcитeтoв: Пилoтнoe Иccлeдoвaниe. Hayкa И Здpaвooxpaнeниe. 2023;5(25):262–269. 10.34689/Sh.2023.25.5.032.

[154] Nada Q, Herdiana I, Andriani F. Testing the validity and reliability of the Depression Anxiety Stress Scale (DASS)-21 instrument for individuals with psychodermatology. Psikohumaniora J Penelitian Psikol. 2022;7(2):153–68.

[155] Malas O, Tolsá MD. Depression, Anxiety and Stress Scales (DASS-21): Factor structure, reliability, invariance and validity of the Catalan Version. Anuario Psicol UB J Psychol. 2022;52(3). https://revistes.ub.edu/index.php/Anuario-psicologia/article/view/37406.

[156] Tsegaye BS, Asemu MM, Hailu HB. Construct validity and reliability of Amharic version of DASS-21 scale among Ethiopian Defense University College of Health Science students. BMC Health Serv Res. 2024;24(1):914. 10.1186/s12913-024-11267-7. 39123213 10.1186/s12913-024-11267-7 PMC11311882

[157] Gibbons FX, Buunk BP. Individual differences in social comparison: development of a scale of social comparison orientation. J Pers Soc Psychol. 1999;76(1):129–42. 10.1037/0022-3514.76.1.129. 9972558 10.1037//0022-3514.76.1.129

[158] Steffen PR, Olsen JA. Conceptualizing personality as individualized allostasis: exploring a balanced measure of personality for psychotherapy/psychophysiology integration. Appl Psychophysiol Biofeedback. 2025;50(2):277–87. 10.1007/s10484-025-09700-6. 40072793 10.1007/s10484-025-09700-6

[159] Diener E, Emmons RA, Larsen RJ, Griffin S. The satisfaction with life scale. J Pers Assess. 1985;49(1):71–5. 10.1207/s15327752jpa4901_13. 16367493 10.1207/s15327752jpa4901_13

[160] Pavot W, Diener E. Review of the satisfaction with life scale. Psychol Assess. 1993;5(2):164.

[161] Avcu A. Item response theory based psychometric investigation of SWLS for university students. Int J Psychol Educ Stud. 2021;8(2):27–37. 10.52380/ijpes.2021.8.2.265.

[162] Glaesmer H, Grande G, Braehler E, Roth M. The german version of the satisfaction with life scale (SWLS): psychometric properties, validity, and population-based norms. Eur J Psychol Assess. 2011;27(2):127–32. 10.1027/1015-5759/a000058.

[163] Rehman S, Alotaibi KA, Rehman E, Khan MN, Rahman MA, Yaqoob B. The buffering effects of mindfulness and organizational support on the mental health of hospital pharmacists in high-workload environments. Sci Rep. 2025;15(1):11994. 40199951 10.1038/s41598-025-96354-3 PMC11978850

[164] Jin Y, Echeverria V, Yan L, Zhao L, Alfredo R, Tsai YS, Gašević D, Martinez-Maldonado R. FATE in MMLA: A student-centred exploration of fairness, accountability, transparency, and ethics in multimodal learning analytics (No. arXiv:2402.19071). arXiv. 2024. 10.48550/arXiv.2402.19071.

[165] Chhibber P, Chahal H, Kaurav RPS. Is brand commitment the missing link in the internal branding and brand citizenship behaviours relationship in services sector: a meta-analysis. Manag Rev Q. 2025;75(2):1259–310. 10.1007/s11301-024-00408-1.

[166] Centofanti F, Hubert M, Rousseeuw PJ. Cellwise and casewise robust covariance in high dimensions (No. arXiv:2505.19925). arXiv. 2025. 10.48550/arXiv.2505.19925.

[167] Revillon G, Djafari A, Enderli C. Variational Bayesian inference for a scale mixture of normal distributions handling missing data (No. arXiv:1711.08374). arXiv. 2017. 10.48550/arXiv.1711.08374.

[168] Gordienko N. Multi-parametric statistical method for estimation of accumulated fatigue by sensors in ordinary gadgets (No. arXiv:1605.04984). arXiv. 2016. 10.48550/arXiv.1605.04984.

[169] Pinsky E, Klawansky S. MAD (about median) vs. quantile-based alternatives for classical standard deviation, skewness, and kurtosis. Front Appl Math Stat. 2023;9:1206537.

[170] Bentler PM, Chou C-P. Practical issues in structural modeling. Sociol Methods Res. 1987;16(1):78–117. 10.1177/0049124187016001004.

[171] Jamshidian M, Jalal S. Tests of homoscedasticity, normality, and missing completely at random for incomplete multivariate data. Psychometrika. 2010;75(4):649–74. 21720450 10.1007/s11336-010-9175-3 PMC3124223

[172] Schafer JL, Graham JW. Missing data: our view of the state of the art. Psychol Methods. 2002;7(2):147. 12090408

[173] Iddrisu AK, Alhassan A. Primary analysis method for incomplete CD4 count data from IMPI trial and other trials with similar setting (No. arXiv:2105.03197). arXiv. 2021. 10.48550/arXiv.2105.03197.

[174] Pandey C, Diwan H. Structural equation modelling to analyze the sustainability of the fertilizer sector in India. Nutr Cycl Agroecosyst. 2025;130(3):347–66. 10.1007/s10705-025-10405-9.

[175] Tong T, Pi F, Zheng S, Zhong Y, Lin X, Wei Y. Exploring the effect of mathematics skills on student performance in physics problem-solving: a structural equation modeling analysis. Res Sci Educ. 2025;55(3):489–509. 10.1007/s11165-024-10201-5.

[176] Mungule CM, Van Vuuren JJ. Validating a measurement scale for entrepreneurial actions for sustainable corporate entrepreneurship using confirmatory factor analysis. Acta Commercii. 2016;16(1):15. 10.4102/ac.v16i1.397.

[177] Sidney P, Braun B, Jong C, Hanely D, Kim M, Brown K, et al. The college mathematics beliefs and belonging survey: instrument development and validation. Int J Res Undergrad Math Educ. 2025;11(2):364–400. 10.1007/s40753-024-00247-1.

[178] Hu L, Bentler PM. Fit indices in covariance structure modeling: sensitivity to underparameterized model misspecification. Psychol Methods. 1998;3(4):424–53. 10.1037/1082-989X.3.4.424.

[179] Ralph P, Baltes S, Adisaputri G, Torkar R, Kovalenko V, Kalinowski M, et al. Pandemic programming: how COVID-19 affects software developers and how their organizations can help. Empir Softw Eng. 2020;25(6):4927–61. 10.1007/s10664-020-09875-y. 32952438 10.1007/s10664-020-09875-y PMC7489196

[180] Kourtesis P, Collina S, Doumas LA, MacPherson SE. Validation of the virtual reality neuroscience questionnaire: maximum duration of immersive virtual reality sessions without the presence of pertinent adverse symptomatology. Front Hum Neurosci. 2019;13:417. 31849627 10.3389/fnhum.2019.00417 PMC6901952

[181] Azarmehr T, Kordbagheri A, Sadat E, Kordbagheri M. Psychometric properties of the Persian version of the Principal Instructional Leadership Scale for High School Principals (PILS): translation, psychometric properties, and network analysis. Asia-Pac Educ Res. 2025;34(3):1197–208. 10.1007/s40299-024-00933-3.

[182] Hair JF, Risher JJ, Sarstedt M, Ringle CM. When to use and how to report the results of PLS-SEM. Eur Bus Rev. 2019;31(1):2–24. 10.1108/EBR-11-2018-0203.

[183] Henseler J, Ringle CM, Sarstedt M. A new criterion for assessing discriminant validity in variance-based structural equation modeling. J Acad Mark Sci. 2015;43(1):115–35. 10.1007/s11747-014-0403-8.

[184] Mussel P. Epistemic curiosity and related constructs: lacking evidence of discriminant validity. Pers Individ Differ. 2010;49(5):506–10.

[185] Hair JF, Ringle CM, Sarstedt M. PLS-SEM: indeed a silver bullet. J Mark Theory Pract. 2011;19(2):139–52. 10.2753/MTP1069-6679190202.

[186] Cui J. The explore of knowledge management dynamic capabilities, AI-driven knowledge sharing, knowledge-based organizational support, and organizational learning on job performance: evidence from chinese technological companies. SSRN.2025. 10.2139/ssrn.5083169.

[187] Jiang H, Yu X. L2 grit and its association with online learning engagement and interaction strategies: a structural equation model. Asia-Pac Educ Res. 2025;34(3):1145–56. 10.1007/s40299-024-00929-z.

[188] Poon K-T, Jiang Y. Getting less likes on social media: mindfulness ameliorates the detrimental effects of feeling left out online. Mindfulness. 2020;11(4):1038–48. 10.1007/s12671-020-01313-w.

[189] Preacher KJ, Rucker DD, Hayes AF. Addressing moderated mediation hypotheses: theory, methods, and prescriptions. Multivar Behav Res. 2007;42(1):185–227. 10.1080/00273170701341316. 10.1080/00273170701341316 26821081

[190] Molnar M. Robust statistical radio interferometric methods for the detection of the epoch of reionization [PhD Thesis]. 2022. https://www.repository.cam.ac.uk/items/06709579-04d6-46c2-95ba-aaa5fc5b6368.

[191] Schumacher FL, Dey DK, Lachos VH. Approximate inferences for nonlinear mixed effects models with scale mixtures of skew-normal distributions. J Stat Theory Pract. 2021;15(3):60. 10.1007/s42519-021-00172-5.

[192] Scherer R, Siddiq F, Tondeur J. The technology acceptance model (TAM): a meta-analytic structural equation modeling approach to explaining teachers’ adoption of digital technology in education. Comput Educ. 2019;128:13–35.

[193] Hanike Y, Damirah D. Modifikasi model analisis Structural Equation Model (Sem) pada Reaksi Pasar di Perusahaan Bursa Efek Indonesia Melalui Modification Indices. Matematika Dan Pembelajaran. 2018;6(2):127–42. 10.33477/mp.v6i2.665.

[194] Jacobsen C, Dong J, Khalloufi M, Huan X, Duraisamy K, Akram M, et al. Enhancing dynamical system modeling through interpretable machine-learning augmentations: a case study in cathodic electrophoretic deposition. Data-Centric Engineering. 2025;6:e4.

[195] Haslbeck J, Borsboom D, Waldorp L. Moderated network models (No. arXiv:1807.02877). arXiv. 2020. 10.48550/arXiv.1807.02877.

[196] Sørensen Ø, Fjell AM, Walhovd KB. Longitudinal modeling of age-dependent latent traits with generalized additive latent and mixed models. Psychometrika. 2023;88(2):456–86. 36976415 10.1007/s11336-023-09910-z PMC10188428

[197] Cheung SF, Cheung S-H. Manymome: an R package for computing the indirect effects, conditional effects, and conditional indirect effects, standardized or unstandardized, and their bootstrap confidence intervals, in many (though not all) models. Behav Res Methods. 2023;56(5):4862–82. 10.3758/s13428-023-02224-z. 37798596 10.3758/s13428-023-02224-z PMC11289038

[198] MacKinnon DP, Lockwood CM, Williams J. Confidence limits for the indirect effect: distribution of the product and resampling methods. Multivar Behav Res. 2004;39(1):99–128. 10.1207/s15327906mbr3901_4 PMC2821115 20157642

[199] Festing MFW. Extending the statistical analysis and graphical presentation of toxicity test results using standardized effect sizes. Toxicol Pathol. 2014;42(8):1238–49. 10.1177/0192623313517771. 24487356 10.1177/0192623313517771

[200] Biscio CAN, Mazoyer A, Vejling MV. Conformal novelty detection for replicate point patterns with FDR or FWER control (No. arXiv:2501.18195). arXiv. 2025. 10.48550/arXiv.2501.18195.

[201] Ma Z, Zeng W. A multiple mediator model: power analysis based on Monte Carlo simulation. Am J Appl Psychol. 2014;3(3):72–9.

[202] Baumann F, Arora N, Rahwan I, Czaplicka A. (Dynamics of algorithmic content amplification on TikTok (No. arXiv:2503.20231). arXiv. 2025. 10.48550/arXiv.2503.20231.

[203] Pin L, Sverdlov O, Bretz F, Bornkamp B. Randomization-based inference for MCP-Mod. Stat Med. 2025;44(10–12):e70092. 10.1002/sim.70092. 40402128 10.1002/sim.70092 PMC12097294

[204] Dalmaijer ES. Tutorial: a priori estimation of sample size, effect size, and statistical power for cluster analysis, latent class analysis, and multivariate mixture models (No. arXiv:2309.00866). arXiv. 2023. 10.48550/arXiv.2309.00866.

[205] Benford S, Mancini C, Chamberlain A, Schneiders E, Castle-Green S, Fischer J, Kucukyilmaz A, Salimbeni G, Ngo V, Barnard P, Adams M, Tandavanitj N. Charting ethical tensions in multispecies technology research through beneficiary-epistemology space. 2024.

[206] Zhu F, Zhang Q, Chen H, Shi G, Wen C, Zhu Z, Chen H. Cardiovascular risk and work stress in biomedical researchers in China: an observational, big data study protocol (No. arXiv:2003.08800). arXiv. 2020. 10.48550/arXiv.2003.08800.

[207] McConnell T. The inalienable right to withdraw from research. J Law Med Ethics. 2010;38(4):840–6. 10.1111/j.1748-720X.2010.00537.x. 21105947 10.1111/j.1748-720X.2010.00537.x

[208] Lee B, Dupervil B, Deputy NP, Duck W, Soroka S, Bottichio L, et al. Protecting privacy and transforming COVID-19 case surveillance datasets for public use. Public Health Reports®. 2021;136(5):554–61. 10.1177/00333549211026817. 34139910 10.1177/00333549211026817 PMC8216038

[209] Sehgal NKR, Kambhamettu H, Matam SP, Ungar L, Guntuku SC. Exploring socio-cultural challenges and opportunities in designing mental health chatbots for adolescents in India. Proceedings of the Extended Abstracts of the CHI Conference on Human Factors in Computing Systems. 2025. 1–7. 10.1145/3706599.3720137.

[210] Park YS, Schroeder D, Kim OJ. Research Ethics Challenges in Pandemic Korea and Their Implications for the Revised 2024 Declaration of Helsinki. J Korean Med Sci. 2025;40(42):e281. 10.3346/jkms.2025.40.e281 10.3346/jkms.2025.40.e281 PMC12591370 41185579

[211] Sharpe D, Ziemer J. Psychology, ethics, and research ethics boards. Ethics Behav. 2022;32(8):658-673. 10.1080/10508422.2021.2023019

[212] Nkenyereye L, Islam SMR, Hossain M, Abdullah-Al-Wadud M, Alamri A. Blockchain-Enabled EHR Framework for Internet of Medical Things (No. arXiv:2011.05935). arXiv. 2020. 10.48550/arXiv.2011.05935.

[213] Hayashi H, Shibanoki T, Tsuji T. A neural network based on the Johnson $S_\mathrm{U}$ translation system and related application to electromyogram classification (No. arXiv:1912.04218). arXiv. 2019. 10.48550/arXiv.1912.04218.

[214] Zuo B, Balakrishnan N, Yin C. An analysis of multivariate measures of skewness and kurtosis of skew-elliptical distributions (No. arXiv:2311.18176). arXiv. 2023. 10.48550/arXiv.2311.18176.

[215] Azur MJ, Stuart EA, Frangakis C, Leaf PJ. Multiple imputation by chained equations: what is it and how does it work? Int J Methods Psychiatr Res. 2011;20(1):40–9. 10.1002/mpr.329. 21499542 10.1002/mpr.329 PMC3074241

[216] Enders C, Bandalos D. The relative performance of full information maximum likelihood estimation for missing data in structural equation models. Struct Equ Model A Multidiscip J. 2001;8(3):430–57. 10.1207/S15328007SEM0803_5.

[217] Ku W, Liu Y, Zhang W, An P. GenPod: constructive news framing in AI-generated podcasts more effectively reduces negative emotions than non-constructive framing (No. arXiv:2412.18300). arXiv. 2024. 10.48550/arXiv.2412.18300.

[218] Kalnins A, Praitis Hill K. Additional caution regarding rules of thumb for variance inflation factors: extending O’Brien to the context of specification error. Qual Quant. 2025;59(S1):291–314. 10.1007/s11135-024-01980-0.

[219] Salmerón R, García C, García J. Overcoming the inconsistences of the variance inflation factor: a redefined VIF and a test to detect statistical troubling multicollinearity (No. arXiv:2005.02245). arXiv. 2020. 10.48550/arXiv.2005.02245.

[220] Gregory B, Peters L. Unique relationships between self-related constructs, social anxiety, and depression in a non-clinical sample. Behav Change. 2017;34(2):117–33.

[221] Zhang Z, Wang Z, Ma T, Taneja VS, Nelson S, Le NHL, Murugesan K, Ju M, Chawla NV, Zhang C, Ye Y. MOPI-HFRS: a multi-objective personalized health-aware food recommendation system with LLM-enhanced interpretation (No. arXiv:2412.08847). arXiv. 2024. 10.48550/arXiv.2412.08847.

[222] Lemos CM, Gore R, Shults FL. Exploratory and confirmatory factor analyses of religiosity. A four-factor conceptual model. PLoS One. 2019;14(5):e0216352. 10.1371/journal.pone.0216352. 31091294 10.1371/journal.pone.0216352 PMC6519809

[223] Rosenblatt JD, Vink M, Benjamini Y. Revisiting multi-subject random effects in fMRI: advocating prevalence estimation. Neuroimage. 2014;84:113–21. 23988271 10.1016/j.neuroimage.2013.08.025

[224] Yao Y, Liu Y, Lu B, Ji G, Wang L, Dong K, et al. Construction and validation of a regulatory T cells-based classification of renal cell carcinoma: an integrated bioinformatic analysis and clinical cohort study. Cell Oncol. 2025;48(3):591–615. 10.1007/s13402-024-01030-9. 10.1007/s13402-024-01030-9 PMC12119669 39714755

[225] Barak S, Landa J, Eisenstein E, Guttman D, Silberg T. Psychometric properties of the Hebrew KIDSCREEN 52, 27 and 10 items: a cross-sectional study of self and parents reports in youth with and without physical disabilities. Qual Life Res. 2025;34(6):1615–31. 10.1007/s11136-025-03941-y. 40080335 10.1007/s11136-025-03941-y

[226] Ahmad N, Du S, Ahmed F, Ul Amin N, Yi X. Healthcare professionals satisfaction and AI-based clinical decision support system in public sector hospitals during health crises: a cross-sectional study. Inf Technol Manag. 2025;26(2):205–17. 10.1007/s10799-023-00407-w.

[227] Choudhury A, Shahsavar Y, Shamszare H. User intent to use DeepSeek for health care purposes and their trust in the large language model: multinational survey study. JMIR Hum Factors. 2025;12:e72867–e72867. 10.2196/72867. 40418796 10.2196/72867 PMC12129370

[228] Ringle CM, Sarstedt M, Mitchell R, Gudergan SP. Partial least squares structural equation modeling in HRM research. Int J Hum Resour Manage. 2020;31(12):1617–43. 10.1080/09585192.2017.1416655.

[229] Ismail K, Nopiah ZM, Mohamad SR, Pang CL. Technical competency among vocational teachers in Malaysian public skills training institutions: measurement model validation using PLS-SEM. J Tech Educ Train. 2020;12(1). https://publisher.uthm.edu.my/ojs/index.php/JTET/article/view/4078.

[230] Guo S, Shi L, Zhai X. Validating an instrument for teachers’ acceptance of artificial intelligence in education (No. arXiv:2406.10506). arXiv. 2024. 10.48550/arXiv.2406.10506.

[231] Tafere Y, Muche AA, Tariku A, Athirsaw A, Alemu K. Cross-cultural adaptation and psychometric evaluation of the child oral health impact profile-short form 19 (COHIP-SF 19) for Ethiopian schoolchildren. BMC Oral Health. 2025;25(1):811. 10.1186/s12903-025-06230-9. 40426099 10.1186/s12903-025-06230-9 PMC12117687

[232] Engelbrecht AS, Heine G, Mahembe B. Integrity, ethical leadership, trust and work engagement. Leadersh Organ Dev J. 2017;38(3):368–79.

[233] Xia Y, Yang Y. RMSEA, CFI, and TLI in structural equation modeling with ordered categorical data: the story they tell depends on the estimation methods. Behav Res Methods. 2019;51(1):409–28. 10.3758/s13428-018-1055-2. 29869222 10.3758/s13428-018-1055-2

[234] Fredrickson BL, Cohn MA, Coffey KA, Pek J, Finkel SM. Open hearts build lives: positive emotions, induced through loving-kindness meditation, build consequential personal resources. J Pers Soc Psychol. 2008;95(5):1045–62. 10.1037/a0013262. 18954193 10.1037/a0013262 PMC3156028

[235] Punjabi P, Jensen A, Ortega AM, Grabow AP. “ Not Liking the Look of This”: examining the relationship between COVID-related social media exposure and mental health symptoms. Psi Chi J Psychol Res. 2024;29(1). https://search.ebscohost.com/login.aspx?direct=true&profile=ehost&scope=site&authtype=crawler&jrnl=21648204&AN=176170451&h=Qehe34pcj0%2BT36X9B1ndUlnlcLybaIVY0PhubcsMuPWArkF9KSVcvTgKxnk1njbpFYMWUWsxoe0Zf4xV00WAIg%3D%3D&crl=c.

[236] Mehta M, Buntain C. Emotional images: assessing emotions in images and potential biases in generative models (No. arXiv:2411.05985). arXiv. 2024. 10.48550/arXiv.2411.05985.

[237] Piccardi T, Saveski M, Jia C, Hancock JT, Tsai JL, Bernstein M. Social media algorithms can shape affective polarization via exposure to antidemocratic attitudes and partisan animosity (No. arXiv:2411.14652). arXiv. 2024. 10.48550/arXiv.2411.14652.

[238] Li WW, Miller D, Leow T, Heward C, Li Y, Yang F, et al. The relationship between mindfulness and mental distress in Chinese people during the COVID-19 pandemic: moderating effects of infection severity of region and mediating effects of resilience and self-efficacy. J Pac Rim Psychol. 2023;17:18344909231192764. 10.1177/18344909231192765.

[239] Zheng J, Hong H, Wang N, Sun J, Xu X. Impact of mindfulness, emotional intelligence, and employee well-being on mental healthcare of workers’ affectiveness: the mediating role of employee satisfaction and the moderating effect of digital leadership. Health Care Anal2025. 10.1007/s10728-025-00523-4. 10.1007/s10728-025-00523-4 40434482

[240] García Del Castillo Rodríguez JA, García Del Castillo López Á, Gázquez Pertusa M, Marzo Campos JC. La Inteligencia Emocional como estrategia de prevención de las adicciones. Health Addictions/Salud Drogas. 2013;13(2):89–97. 10.21134/haaj.v13i2.204.

[241] Teodorescu D, Cheng T, Fyshe A, Mohammad SM. Language and mental health: measures of emotion dynamics from text as linguistic biosocial markers (No. arXiv:2310.17369). arXiv. 2023. 10.48550/arXiv.2310.17369.

[242] Maltese F, Pacinelli G, Monai A, Bernardi F, Capaz AM, Niello M, et al. Self-experience of a negative event alters responses to others in similar states through prefrontal cortex CRF mechanisms. Nat Neurosci. 2025;28(1):122–36. 39627538 10.1038/s41593-024-01816-y

[243] Santoso B. The impact of social media use on soldier morale: the mediating role of psychological resilience and moderating effect of mission length. Stud Media Commun. 2025;13(3):49–61.

[244] Robinson NL, Connolly J, Suddrey G, Kavanagh DJ. A brief wellbeing training session delivered by a humanoid social robot: a pilot randomized controlled trial. Int J Soc Robot. 2024;16(5):937–51. 10.1007/s12369-023-01054-5.

[245] Salathé M, Vu DQ, Khandelwal S, Hunter DR. The dynamics of health behavior sentiments on a large online social network. EPJ Data Sci. 2013;2(1):4. 10.1140/epjds16.

[246] Alshamsi A, Pianesi F, Lepri B, Pentland A, Rahwan I. Beyond contagion: reality mining reveals complex patterns of social influence. PLoS One. 2015;10(8):e0135740. 10.1371/journal.pone.0135740. 26313449 10.1371/journal.pone.0135740 PMC4551670

[247] Lo IPY, Kim YK, Liu EH, Yan E. Typologies of minority stressors and depressive symptoms among LGBTQ employees in the workplace: a moderated mediation model of workplace climate and resilience. Sex Res Soc Policy. 2025;22(2):1043–57. 10.1007/s13178-024-01027-x.

[248] Pauly C, Ribeiro F, Schröder VE, Pauly L, Krüger R, Leist AK, et al. The moderating role of resilience in the personality-mental health relationship during the COVID-19 pandemic. Front Psychiatry. 2021;12:745636. 34744837 10.3389/fpsyt.2021.745636 PMC8566705

[249] Karinta A. Negative effects of social media use on mental health in adolescents. Media Gizi Kesmas. 2022;11(1):307–12.

[250] Funk S. Sick of leading? Supervisory responsibility and its consequences for sickness absenteeism and sickness presenteeism. J Bus Psychol. 2025;40(3):651–67. 10.1007/s10869-024-09980-5.

[251] Jamerson KA, Nesbitt SD, Amerena JV, Grant E, Julius S. Angiotensin mediates forearm glucose uptake by hemodynamic rather than direct effects. Hypertension. 1996;27(4):854–8. 10.1161/01.HYP.27.4.854. 8613260 10.1161/01.hyp.27.4.854

[252] Catalogna M, Somerville Y, Saporta N, Nathansohn-Levi B, Shelly S, Edry L, et al. Brain connectivity correlates of the impact of a digital intervention for individuals with subjective cognitive decline on depression and IL-18. Sci Rep. 2025;15(1):6863. 40011544 10.1038/s41598-025-91457-3 PMC11865443

[253] Maheswaran H, Weich S, Powell J, Stewart-Brown S. Evaluating the responsiveness of the Warwick Edinburgh Mental Well-Being Scale (WEMWBS): group and individual level analysis. Health Qual Life Outcomes. 2012;10(1):156. 10.1186/1477-7525-10-156. 23270465 10.1186/1477-7525-10-156 PMC3560098

[254] Maloney SJ, Richards J, Nixon DGD, Harvey LJ, Fletcher IM. Do stiffness and asymmetries predict change of direction performance? J Sports Sci. 2016. 1–10. 10.1080/02640414.2016.1179775. 10.1080/02640414.2016.1179775 27133586

[255] Cohen J. Statistical power analysis for the behavioral sciences. Routledge. 2013. https://www.taylorfrancis.com/books/mono/10.4324/9780203771587/statistical-power-analysis-behavioral-sciences-jacob-cohen.

[256] Feruglio S, Panasiti MS, Crescentini C, Aglioti SM, Ponsi G. Training the moral self: an 8-week mindfulness meditation program leads to reduced dishonest behavior and increased regulation of interoceptive awareness. Mindfulness. 2023;14(11):2757–79. 10.1007/s12671-023-02233-1.

[257] Laban G, Wang J, Gunes H. A Robot-led intervention for emotion regulation: from expression to reappraisal (No. arXiv:2503.18243). arXiv. 2025. 10.48550/arXiv.2503.18243.

[258] Rahman A, Syeed MMM, Karim MR, Fatema K, Khan RH, Uddin MF. An optimized ensemble ML-WQI model for reliable water quality prediction by minimizing the eclipsing and ambiguity issues. Appl Water Sci. 2025;15(5):113. 10.1007/s13201-025-02450-0.

[259] Wilson M, Needham T, Srivastava A. Fused gromov-wasserstein variance decomposition with linear optimal transport (No. arXiv:2411.10204). arXiv. 2024. 10.48550/arXiv.2411.10204.

[260] Alluhidan A, Akter M, Alsoubai A, Park JK, Wisniewski P. Teen talk: the good, the bad, and the neutral of adolescent social media use. Proc ACM Hum Comput Interact. 2024;8(CSCW2):1–35. 10.1145/3686961.39286336

[261] Madraki G, Grasso I, M. Otala J, Liu Y, Matthews J. Characterizing and comparing COVID-19 misinformation across languages, countries and platforms. Companion Proceedings of the Web Conference. 2021. 213–223. 10.1145/3442442.3452304.

[262] Gabarron E, Larbi D, Dorronzoro E, Hasvold PE, Wynn R, Årsand E. Factors engaging users of diabetes social media channels on Facebook, Twitter, and Instagram: observational study. J Med Internet Res. 2020;22(9):e21204. 10.2196/21204. 32990632 10.2196/21204 PMC7556374

[263] Liotsiou D, Ganesh B, Howard PN. Predicting Engagement with the Internet Research Agency’s Facebook and Instagram Campaigns around the 2016 U.S. Presidential Election (No. arXiv:2010.14950). 2020. arXiv. 10.48550/arXiv.2010.14950.

[264] Annabell T, Gorwa R, Scharlach R, van de Kerkhof J, Bertaglia T. TikTok Search Recommendations: Governance and Research Challenges (No. arXiv:2505.08385). arXiv. 2025. 10.48550/arXiv.2505.08385.

[265] Zeng J, Kaye DBV. From content moderation to visibility moderation: a case study of platform governance on TikTok. Policy Internet. 2022;14(1):79–95. 10.1002/poi3.287.

[266] Sharma P, Chen IS, Luk ST. Gender and age as moderators in the service evaluation process. J Serv Mark. 2012;26(2):102–14.

[267] Xie H, Ouyang H, Yaacob NRN, Feng D, Wang S. Challenges and opportunities in teaching through interactions framework: a three-level meta-analysis of the CLASS measure and children’s vocabulary skills. Early Child Educ J. 2025;53(5):1605–25. 10.1007/s10643-024-01703-y.

[268] Evans O, Hardacre S, Rubin M, Tran M. Content appraisal and age moderate the relationship between passive social media use and mental ill-being. Front Psychol. 2023;14:1181233. 37529318 10.3389/fpsyg.2023.1181233 PMC10388548

[269] Pochwatko G, Karpowicz B, Chrzanowska A, Kopeć W. Interpersonal distance in VR: reactions of older adults to the presence of a virtual agent. In Biele C, Kacprzyk J, Owsiński JW, Romanowski A. Sikorski M (Eds.). Digital Interaction and Machine Intelligence (Vol. 1376). Springer International Publishing. 2021. pp. 91–100. 10.1007/978-3-030-74728-2_9.

[270] Medina-Garrido JA, Biedma-Ferrer JM, Ramos-Rodríguez AR. Moderating effects of gender and family responsibilities on the relations between work–family policies and job performance. Int J Hum Resour Manage. 2021;32(5):1006–37. 10.1080/09585192.2018.1505762.

[271] Powell LA. The associations between overweight/obesity among children and select social and economic predictors. 2022.

[272] Li J, Cochrane KA, Leshed G. Beyond meditation: understanding everyday mindfulness practices and technology use among experienced practitioners. Proc ACM Hum Comput Interact. 2024;8(CSCW2):1–29. 10.1145/3687023.39286336

[273] Montes CM, Sjögren F, Klevfors A, Penzenstadler B. Qualifying and quantifying the benefits of mindfulness practices for IT workers. 10th International Conference on ICT for Sustainability (ICT4S). 2024. 272–281. https://ieeexplore.ieee.org/abstract/document/10805310/.

[274] Dominguez-Catena I, Paternain D, Galar M. Gender stereotyping impact in facial expression recognition. 2023;1752:9–22. 10.1007/978-3-031-23618-1_1.

[275] Raman V, Fleisig E, Klein D. Centering the Margins: outlier-based identification of harmed populations in toxicity detection (No. arXiv:2305.14735). arXiv. 2023. 10.48550/arXiv.2305.14735.

[276] Tartler D, Handke L, Kauffeld S. Designing virtual meetings: reviewing virtual meeting design through the lens of media naturalness. Electron Markets. 2025;35(1):41. 10.1007/s12525-025-00789-5.

[277] Jarrahi MH, Blyth DL, Goray C. Mindful work and mindful technology: redressing digital distraction in knowledge work. Digital Business. 2023;3(1):100051.

[278] Deroche M-H, Kuyken W, Uwatoko T, Imoto Y, Kusumoto R. The mindful way from information to knowledge, to wisdom, and to life: perspectives on mindfulness (-based cognitive therapy) for higher education. Mindfulness. 2025;16(4):846–63. 10.1007/s12671-025-02528-5. 40230594 10.1007/s12671-025-02528-5 PMC11993468

[279] Fischer R, Karl JA, Daly A, Bortolini G. One with Nature, One with Each Other? A niche construction framework linking rituals, social media and nature connectedness. Topoi. 2025. 10.1007/s11245-025-10189-1.

[280] McManus J. Emotions and ethical decision making at work: organizational norms, emotional dogs, and the rational tales they tell themselves and others. J Bus Ethics. 2021;169(1):153–68. 10.1007/s10551-019-04286-6.

[281] Wu J, Peng H. Posting short-form videos promotes older adults’ psychological health: a model based on self-determination theory. Innov Aging. 2024;8(Supplement_1):1102–1102.

[282] Holtz D, Carterette B, Chandar P, Nazari Z, Cramer H, Aral S. The engagement-diversity connection: evidence from a field experiment on spotify. Proceedings of the 21st ACM Conference on Economics and Computation. 2020. 75–76. 10.1145/3391403.3399532.

[283] Xuan W, Chowdhury MR, Ding Y, Zhao Y. Unlocking mental health: exploring college students’ well-being through smartphone behaviors (No. arXiv:2502.08766). arXiv. 2025. 10.48550/arXiv.2502.08766.

[284] Mitsea E, Drigas A, Skianis C. Brain-computer interfaces in digital mindfulness training for metacognitive, emotional and attention regulation skills: a literature review. Res Soc Dev. 2023;12(3):e2512340247–e2512340247.

[285] Rasch J, Zender MJ, Sakel S, Wagener N. Mind mansion: exploring metaphorical interactions to engage with negative thoughts in virtual reality. designing interactive systems conference. 2024. 2305–2318. 10.1145/3643834.3661557.

[286] Apolinário-Hagen J, Drüge M, Guthardt L, Haller E. Acceptance and commitment therapy for major depressive disorder: navigating depression treatment in traditional and digital settings with insights from current research. In Y.-K. Kim (Ed.), Recent Advances and Challenges in the Treatment of Major Depressive Disorder (Vol. 1456). Springer Nature Singapore; 2024. pp. 227–256. 10.1007/978-981-97-4402-2_12. 10.1007/978-981-97-4402-2_12 39261432

[287] Long KS. Understanding, measuring, and, invoking mindfulness and mindlessness during human-computer interactions. Lancaster University (United Kingdom). 2018. https://search.proquest.com/openview/f3ef9720b73c2bc6354d24b90a976d18/1?pq-origsite=gscholar&cbl=51922&diss=y.

[288] Shen JJ, King Chen J, Findlater L, Dietz Smith G. eaSEL: promoting social-emotional learning and parent-child interaction through AI-mediated content consumption. Proceedings of the 2025 CHI Conference on Human Factors in Computing Systems. 2025. 1–18. 10.1145/3706598.3713405.

[289] Mohammed H. Technology in Association With Mental Health: Meta-ethnography (No. arXiv:2307.10513). arXiv. 2023. 10.48550/arXiv.2307.10513

[290] Rabbi M, Philyaw-Kotov M, Li J, Li K, Rothman B, Giragosian L, Reyes M, Gadway H, Cunningham R, Bonar E, Nahum-Shani I, Walton M, Murphy S, Klasnja P. Translating behavioral theory into technological interventions: case study of an mHealth app to increase self-reporting of substance-use related data (No. arXiv:2003.13545). arXiv. 2020. 10.48550/arXiv.2003.13545.

[291] Gourabi S, Khosravani P, Nosrat S, Mohammadi R, Lotfalipour M. Network dynamics of emotional processing: a structural balance theory approach (No. arXiv:2412.09554). arXiv. 2024. 10.48550/arXiv.2412.09554.

[292] Dhelim S, Chen LL, Ning H, Das SK, Nugent C, Burns D, Leavey G, Pesch D, Bantry-White E. Social behavior and mental health: a snapshot survey under COVID-19 pandemic (No. arXiv:2105.08165). arXiv. 2021. 10.48550/arXiv.2105.08165.

[293] Ye T, Yan H, Huang X, Grogan C, Yuan W, Mei Q, Jackson MO. Content quality vs. attention allocation: an LLM-based case study in peer-to-peer mental health networks (No. arXiv:2411.05328). arXiv. 2024. 10.48550/arXiv.2411.05328.

[294] Baer RA. Measuring mindfulness. Contemp Buddhism. 2011;12(1):241–61. 10.1080/14639947.2011.564842.

[295] Ghayoumi M, Ghazinour K, Ghayoumi M. Early Alzheimer’s detection using bidirectional LSTM and attention mechanisms in eye tracking. In Hodson DD, Grimaila MR, Arabnia HR, Deligiannidis L, Wagner TJ (Eds.). Scientific computing and bioinformatics and computational biology (Vol. 2258). Springer Nature Switzerland. 2025. pp. 295–312. 10.1007/978-3-031-85902-1_26.

[296] Moser JS, Hajcak G, Bukay E, Simons RF. Intentional modulation of emotional responding to unpleasant pictures: an ERP study. Psychophysiology. 2006;43(3):292–6. 10.1111/j.1469-8986.2006.00402.x. 16805868 10.1111/j.1469-8986.2006.00402.x

[297] Saldias FB, Picard RW. Tweet Moodifier: Towards giving emotional awareness to Twitter users. 2019 8th International Conference on Affective Computing and Intelligent Interaction (ACII). 2019. 1–7. https://ieeexplore.ieee.org/abstract/document/8925533/.

[298] Tajmirriyahi M, Ickes W. Evidence that increasing self-concept clarity tends to reduce the role of emotional contagion in predicting one’s emotional intelligence regarding a romantic partner. Pers Individ Differ. 2022;185:111259.

[299] Chehayeb L, Bhuvaneshwara C, Anglet M, Hilpert B, Meyer AK, Tsovaltzi D, Gebhard P, Biermann A, Auchtor S, Lauinger N, Knopf J, Kaiser A, Kersting F, Mehlmann G, Lingenfelser F, André E. MITHOS: interactive mixed reality training to support professional socio-emotional interactions at schools (No. arXiv:2409.12968). arXiv. 2024. 10.48550/arXiv.2409.12968.

[300] Skeggs A, Orben A. Social media interventions to improve well-being. Nat Hum Behav. 2025;9:1079–1089. 10.1038/s41562-025-02167-9 40374729

[301] Martel-Santana A, Martín-del-Pozo M. A usability evaluation of a serious game for tackling bullying and cyberbullying in primary education by pre-service teachers. Technol Knowl Learn2025. 10.1007/s10758-025-09850-w.

[302] Rutherford MW, Whitacre BE, Captain L, Ekin S, Angle J, Hensley T, O’Hara JF. Promoting rural entrepreneurship through technology: a case study using Productivity Enhancing Technology Experience Kits (PETE-Kits). IEEE Transact Educ2025. 10.1109/TE.2025.3557023.

[303] Nie M. Algorithmic addiction by design: big tech’s leverage of dark patterns to maintain market dominance and its challenge for content moderation (No. arXiv:2505.00054). arXiv. 2025. 10.48550/arXiv.2505.00054.

[304] Rahmani AM, Lai J, Jafarlou S, Azimi I, Yunusova A, Rivera AP, et al. Personal mental health navigator: harnessing the power of data, personal models, and health cybernetics to promote psychological well-being. Front Digit Health. 2022;4:933587. 36213523 10.3389/fdgth.2022.933587 PMC9535086

[305] Sajid S, Anwer M, Mufti AA, Iqbal M. Investigating how cultural contexts shape social media experiences and their emotional consequence. Rev Educ Adm Law. 2024;7(4):185–200. 10.47067/real.v7i4.370.

[306] Wan Q, Feng X, Bei Y, Gao Z, Lu Z. Metamorpheus: interactive, affective, and creative dream narration through metaphorical visual storytelling. Proceedings of the CHI Conference on Human Factors in Computing Systems. 2024. 1–16. 10.1145/3613904.3642410.

[307] Muzumdar P, Cheemalapati S, RamiReddy SR, Singh K, Kurian G, Muley A. The dead internet theory: a survey on artificial interactions and the future of social media. Asian J Res Comput Sci. 2025;18(1):67–73. 10.9734/ajrcos/2025/v18i1549.

[308] Epstein Z, Lin H, Pennycook G, Rand D. How many others have shared this? Experimentally investigating the effects of social cues on engagement, misinformation, and unpredictability on social media (No. arXiv:2207.07562). arXiv. 2022. 10.48550/arXiv.2207.07562.

[309] Nirmal A, Jiang B, Liu H. SocioHub: An interactive tool for cross-platform social media data collection (No. arXiv:2309.06525). arXiv. 2023. 10.48550/arXiv.2309.06525.

[310] Chen T, Chen Y, Luo J. A selfie is worth a thousand words: mining personal patterns behind user selfie-posting behaviours. Proceedings of the 26th International Conference on World Wide Web Companion - WWW ’17 Companion. 2017. 23–31. 10.1145/3041021.3054142.

[311] Manikonda L, Meduri VV, Kambhampati S. Tweeting the mind and instagramming the heart: Exploring differentiated content sharing on social media. Proc Inte AAAI Conf Web Soc Media. 2016;10(1):639–42. https://ojs.aaai.org/index.php/ICWSM/article/view/14819.

[312] Ziat M, Jhunjhunwala R, Gharat A, Deshpande Y, Raisamo R. Levitation experience in virtual reality. Virtual Reality. 2025;29(2):68. 10.1007/s10055-025-01138-9.

[313] Cooper DM. Needs, passions and loot boxes—exploring reasons for problem behaviour in relation to loot box engagement (No. arXiv:2307.04549). arXiv. 2023. 10.48550/arXiv.2307.04549.

[314] Wu Y, Qin L, Xu X, Tian Y, Jia Z. Dual pathways linking mindfulness to life satisfaction and depression: the mediating roles of self-compassion and rumination in Chinese university students. BMC Psychol. 2025;13(1):570. 10.1186/s40359-025-02895-7. 40426204 10.1186/s40359-025-02895-7 PMC12117703

[315] Wang X, Mo X, Fan M, Lee LH, Shi B, Hui P. Reducing stress and anxiety in the metaverse: a systematic review of meditation, mindfulness and virtual reality. Proceedings of the Tenth International Symposium of Chinese CHI. 2022. 170–180. 10.1145/3565698.3565781.

[316] Bowman NA, Hill PL. Measuring how college affects students: Social desirability and other potential biases in college student self-reported gains. New Dir Inst Res. 2011;2011(150):73–85. 10.1002/ir.390.

[317] Kuentzel JG, Henderson MJ, Melville CL. The impact of social desirability biases on self-report among college student and problem gamblers. J Gambl Stud. 2008;24(3):307–19. 10.1007/s10899-008-9094-8. 18369710 10.1007/s10899-008-9094-8

[318] Alvari G, Vallefuoco E, Cristofolini M, Salvadori E, Dianti M, Moltani A, Castello DD, Venuti P, Furlanello C. Exploring physiological responses in virtual reality-based interventions for autism spectrum disorder: a data-driven investigation (Version 1). arXiv. 2024. 10.48550/ARXIV.2404.07159.

[319] Gamez D, Barcari D, Grig A. A new type of foundation model based on recordings of People’s emotions and physiology (No. arXiv:2408.00030). arXiv. 2024. 10.48550/arXiv.2408.00030.

[320] Gansner M, Horton AK, Singh R, Schuman-Olivier Z. Exploring relationships between social media use, online exposure to drug-related content, and youth substance use in real time: a pilot ecological momentary assessment study in a clinical sample of adolescents and young adults. Front Child Adolesc Psychiatry. 2024;3:1369810. 10.3389/frcha.2024.1369810. 39834351 10.3389/frcha.2024.1369810 PMC11745144

[321] Teng S, D’Alfonso S, Kostakos V. Understanding user behavior in the wild using smartphones. In Vanderdonckt J, Palanque P, Winckler M. (Eds.). Handbook of Human Computer Interaction. Springer Nature Switzerland. 2025. pp. 1–26. 10.1007/978-3-319-27648-9_109-1.

[322] Mushtaq A, Naeem MR, Taj MI, Ghaznavi I, Qadir J. Toward inclusive educational AI: auditing frontier LLMs through a Multiplexity Lens (No. arXiv:2501.03259). arXiv. 2025. 10.48550/arXiv.2501.03259.

[323] Seo J, Ammari T. Pragmatic disengagement and culturally situated non use older Korean immigrants strategies for navigating digital noise (No. arXiv:2505.18326). arXiv. 2025. 10.48550/arXiv.2505.18326.

[324] Shahwar D. The effectiveness of social media in enhancement of news consumption. UW J Soc Sci. 2024;7(2):70–81.

[325] Silveira P. Fake news consumption through social media platforms and the need for media literacy skills: A real challenge for Z Generation. INTED2020 Proceedings. 2020. 3830–3838. https://library.iated.org/view/SILVEIRA2020FAK.

[326] Meske C, Amojo I. Status Quo, critical reflection and road ahead of digital nudging in information systems research—a discussion with markus weinmann and alexey voinov. Commun Assoc Inform Syst. 2020. 402–420. 10.17705/1CAIS.04617.

[327] Hu T, Collier N. iNews: a multimodal dataset for modeling personalized affective responses to news (No. arXiv:2503.03335). arXiv. 2025. 10.48550/arXiv.2503.03335.

[328] Mathes J, Schuffelen J, Gieselmann A, Pietrowsky R. Nightmare distress is related to traumatic childhood experiences, critical life events and emotional appraisal of a dream rather than to its content. J Sleep Res. 2023;32(3):e13779. 10.1111/jsr.13779. 36333940 10.1111/jsr.13779

[329] Elahimanesh S, Mohammadkhani M, Kasaei S. Emotion alignment: discovering the gap between social media and real-world sentiments in persian tweets and images (No. arXiv:2504.10662). arXiv. 2025. 10.48550/arXiv.2504.10662.

[330] Ohme J, Araujo T, Boeschoten L, Freelon D, Ram N, Reeves BB, et al. Digital trace data collection for social media effects research: APIs, data donation, and (screen) tracking. Commun Methods Meas. 2024;18(2):124–41. 10.1080/19312458.2023.2181319.

[331] Hoepfel D, Bila A, Günther V, Kersting A, Suslow T. Attention to facial emotions in adult women varies by type and severity of childhood maltreatment experience and emotion regulation strategy. Sci Rep. 2025;15(1):16266. 10.1038/s41598-025-99562-z. 40346180 10.1038/s41598-025-99562-z PMC12064654

[332] Pang D, Van Woerkom M. Enhancing knowledge workers’ well-Being and productivity: a mindful co-working design approach. Gruppe. Interaktion. Organisation. Zeitschrift Für Angewandte Organisationspsychologie (GIO). 2025.

[333] Yildirim C, O’Grady T. The efficacy of a virtual reality-based mindfulness intervention. 2020 IEEE International Conference on Artificial Intelligence and Virtual Reality (AIVR). 2020. 158–165. https://ieeexplore.ieee.org/abstract/document/9319114/.

[334] Maksatbekova A, Argan M. Virtual reality’s dual edge: navigating mental health benefits and addiction risks across timeframes. Curr Psychol. 2025;44(7):6469–80. 10.1007/s12144-025-07623-3.

[335] Wohlfart O, Wagner I. Longitudinal perspectives on technology acceptance: teachers’ integration of digital tools through the COVID-19 transition. Educ Inf Technol. 2025;30(5):6091–115. 10.1007/s10639-024-12954-y.

[336] Peters D. Wellbeing supportive design – research-based guidelines for supporting psychological wellbeing in user experience. Int J Human-Comput Interact. 2023;39(14):2965–77. 10.1080/10447318.2022.2089812.

[337] Berkovich-Ohana A, Brown KW, Gallagher S, Barendregt H, Bauer P, Giommi F, et al. Pattern theory of selflessness: how meditation may transform the self-pattern. Mindfulness. 2024;15(8):2114–40. 10.1007/s12671-024-02418-2.

[338] Guendelman S, Kaltwasser L, Bayer M, Gallese V, Dziobek I. Brain mechanisms underlying the modulation of heart rate variability when accepting and reappraising emotions. Sci Rep. 2024;14(1):18756. 39138266 10.1038/s41598-024-68352-4 PMC11322180

[339] Jiang X, Chen Y, Pal NR, Chang YC, Yang Y, Do T, Lin CT. Interpretable Dual-Filter Fuzzy Neural Networks for Affective Brain-Computer Interfaces (No. arXiv:2502.17445). arXiv. 2025. 10.48550/arXiv.2502.17445.

[340] Monachesi B, Grecucci A, Ghomroudi PA, Messina I. Understanding the neural architecture of emotion regulation by comparing two different strategies: a meta-analytic approach (No. arXiv:2305.16241). arXiv. 2023. 10.48550/arXiv.2305.16241.

[341] Shaayesteh MT, Esfahani SM, Mohit H. AI identity, empowerment, and mindfulness in mitigating unethical AI use (No. arXiv:2503.20099). arXiv. 2025. 10.48550/arXiv.2503.20099.

[342] Choi JI, Agichtein E. Quantifying the effects of prosody modulation on user engagement and satisfaction in conversational systems. Proceedings of the 2020 Conference on Human Information Interaction and Retrieval. 2020. 417–421. 10.1145/3343413.3378009.

[343] Konyspay A, Shamoi P, Ziyada M, Smambayev Z. Meme similarity and emotion detection using multimodal analysis (No. arXiv:2503.17493). arXiv. 2025. 10.48550/arXiv.2503.17493.

[344] Nordahl H, Plummer A, Wells A. Predictors of biased self-perception in individuals with high social anxiety: the effect of self-consciousness in the private and public self domains. Front Psychol. 2017;8:1126. 28725207 10.3389/fpsyg.2017.01126 PMC5495823

[345] Panchanadikar R. Exploring proactive interventions toward harmful behavior in embodied virtual spaces (No. arXiv:2405.05920). arXiv. 2024. 10.48550/arXiv.2405.05920.

