# Supplementary material for: Digital emotional regulation paradox: a cross-sectional study on mindful technology use moderates the relationship between social media emotional content exposure and psychological resilience
Source: BMC Psychol. 2025 Nov 25;13:1411. doi: 10.1186/s40359-025-03727-4 (PMC12751459; doi:10.1186/s40359-025-03727-4)
Supplement: Supplementary file 1 — Supplementary Material 1 [file 40359_2025_3727_MOESM1_ESM.pdf]

Supplementary Material 1
Newly Developed Measurement Instruments

Associated with the article: **Digital Emotional Regulation Paradox: How Mindful Technology Use Moderates the Relationship Between Social Media Emotional Content Exposure and Psychological Resilience**

A. Social Media Emotional Content Exposure (SMECE) Scale

Description

The SMECE Scale was developed for this study to assess participants' exposure to emotionally charged content on social media platforms. It includes 18 items divided into three subscales: Positive Emotional Content Exposure (6 items), Negative Emotional Content Exposure (6 items), and Mixed/Neutral Emotional Content Exposure (6 items).

Instructions to Respondents

Please think about your typical social media use (e.g., Instagram, TikTok, Facebook, X/Twitter, YouTube). Indicate how often and how strongly you are exposed to posts or content with the emotional characteristics described below.

Respond using the following scale: 1 = Never, 7 = Very Frequently (for frequency items); 1 = Not at all intense, 7 = Extremely intense (for intensity items).

Items

Positive Emotional Content Exposure

1. How often do you encounter posts that make you feel inspired or uplifted?
2. How frequently do you see content that conveys happiness or gratitude?
3. How emotionally intense are the positive posts you typically see?
4. How often do you come across supportive or encouraging comments?
5. How emotionally powerful are the joyful or humorous posts you encounter?
6. How often do you interact with positive news or success stories online?

Negative Emotional Content Exposure

1. How often do you encounter posts that make you feel sad or upset?
2. How frequently do you see content that expresses anger or frustration?
3. How emotionally intense are the negative posts you typically see?
4. How often do you come across distressing or conflict-based online discussions?
5. How often do you encounter anxiety-provoking or fear-inducing content?
6. How emotionally intense are the posts that convey loss, conflict, or outrage?

Mixed/Neutral Emotional Content Exposure

1. How often do you see content that presents both positive and negative perspectives on the same topic?
2. How frequently do you encounter posts that leave you feeling emotionally conflicted?

3. How often do you encounter neutral or balanced discussions of emotionally charged issues?
4. How emotionally complex do you find posts that combine humor with critique or sarcasm?
5. How often do you interact with content that mixes emotional tones (e.g., bittersweet posts)?
6. How emotionally engaging are the posts that express both appreciation and disappointment?

B. Mindful Technology Use (MTU) Scale

Description

The MTU Scale was developed for this study to measure the extent to which individuals use digital technology intentionally and with awareness of its emotional and psychological impact. It consists of 24 items across four subscales: Intentional Engagement (6 items), Digital Awareness (6 items), Technology–Life Balance (6 items), and Values Alignment (6 items).

Instructions to Respondents

Please read each statement carefully and indicate how much you agree or disagree, based on how you generally interact with technology and social media.

Respond using the following scale: 1 = Strongly Disagree, 7 = Strongly Agree.

Items

Intentional Engagement

1. I consciously decide when to check social media rather than doing it automatically.
2. I pause and reflect before responding to emotionally charged posts.
3. I regularly evaluate whether my online activity aligns with my current goals.
4. I make deliberate choices about how long to spend online.
5. I am aware of the emotional tone of my online interactions.
6. I intentionally schedule “tech-free” times during my day.

Digital Awareness

1. I notice how different types of online content affect my mood.
2. I can recognize when my online activity increases my stress or anxiety.
3. I am attentive to how my body feels when I spend long periods online.
4. I observe my emotional reactions while scrolling through social media.
5. I notice when I’m using technology as a distraction from difficult emotions.
6. I am aware of how my attention shifts during online multitasking.

Technology–Life Balance

1. I maintain clear boundaries between my online and offline time.
2. I make time for offline relationships even when online interactions are available.
3. I limit notifications that interrupt my focus.
4. I prioritize sleep and rest over late-night social media use.
5. I avoid letting technology use interfere with face-to-face communication.

6. I balance digital engagement with physical and social activities.

Values Alignment

1. My technology use aligns with my personal values and priorities.
2. I use social media to support causes that are meaningful to me.
3. I avoid online behaviors that conflict with my ethical beliefs.
4. I choose platforms that reflect my values regarding privacy and wellbeing.
5. I seek online communities that foster kindness and respect.
6. I use technology to express gratitude and empathy toward others.

Scoring and Interpretation

Higher scores indicate greater mindful awareness and intentionality in technology use. Subscale scores can be averaged individually, or summed to generate a total MTU score. Both SMECE and MTU demonstrated strong internal consistency ($\alpha > .85$) and construct validity based on exploratory and confirmatory factor analyses.

Citation

Sandra, L. (2025). Supplementary Material 1 – Newly Developed Measurement Instruments (SMECE and MTU Scales). In Digital Emotional Regulation Paradox: How Mindful Technology Use Moderates the Relationship Between Social Media Emotional Content Exposure and Psychological Resilience. BMC Psychology.
